# Oxidized MIF is an Alzheimer’s disease drug target relaying external risk factors to tau pathology

**DOI:** 10.1016/j.xcrm.2025.102520

**Published:** 2025-12-18

**Authors:** Andreas Müller-Schiffmann, Felix Torres, Anatoliy Kitaygorodskyy, Anand Ramani, Argyro Alatza, Sarah K. Tschirner, Julien Orts, Arthur Haltrich, Ingrid Prikulis, Shaofeng Yu, Debendranath Dey, Suguna Mallesh, Dharma Prasad, Dennis Solas, Verian Bader, Annemieke Rozemuller, Selina Wray, Jay Gopalakrishnan, Roland Riek, Vishwanath R. Lingappa, Carsten Korth

**Affiliations:** 1 Department Neuropathology, Heinrich Heine University Düsseldorf, 40225 Düsseldorf, Germany; 2 Laboratory of Physical Chemistry, ETH Zürich, Vladimir Prelog Weg 2, 8093 Zürich, Switzerland; 3 Prosetta Biosciences Inc, 670 5th Street, San Francisco, CA 94107, USA; 4 Institute of Human Genetics, University Hospital, Friedrich-Schiller-Universität Jena, 07740 Jena, Germany; 5 Department of Neurodegenerative Disease, UCL Queen Square Institute of Neurology, London WC1N 1PJ, UK; 6 Department Pathology, VUMC Amsterdam, 1081HV Amsterdam, the Netherlands; 7 Department of Pharmaceutical Sciences, University of Vienna, 1090 Vienna, Austria

**Keywords:** oxMIF, macrophage migration inhibitory factor, Alzheimer’s disease, tau, protein aggregation, herpes virus, risk factor, proteostasis, tauopathy, neurodegeneration

## Abstract

During deep co-evolution of viruses and host cells, viruses have selected specific host cellular proteins redirected from physiological functions to viral needs, thereby disturbing cellular proteostasis and increasing the risk of triggering protein misfolding diseases (PMDs). Identifying virus-specific, repurposed host proteins also allows the study of fundamental cellular events in “sporadic” PMDs, independent of the virus. Here, we identify a small molecule with very strong activity against neurotropic herpes simplex virus 1 (HSV-1), modulating an allosteric site of macrophage migration inhibitory factor (MIF). The compound efficiently reduces both HSV-1-mediated and non-mediated tau phosphorylation or aggregation *in vitro* and *in vivo*. The lead compound, as well as conformation-sensitive antibodies, specifically interacts with an oxidized conformer of MIF (oxMIF) enriched in *postmortem* brain homogenates of patients with Alzheimer’s disease (AD). OxMIF thus participates in a host-viral interface connecting HSV-1 infection, and possibly other external stressors, with tau cellular pathology characteristic for PMDs, including AD.

## Highlights

• HSV-1 replication involves oxMIF and can be blocked by PAV-174 inhibiting oxMIF
• OxMIF, detected by a specific antibody, is enriched in brain of patients with sAD
• PAV-174/ PAV-617 inhibit oxMIF-induced tau phosphorylation *in vitro* and *in vivo* independent of infection
• OxMIF is a molecular interface between external stressors and cellular AD pathology

## Graphical abstract

**Figure:**
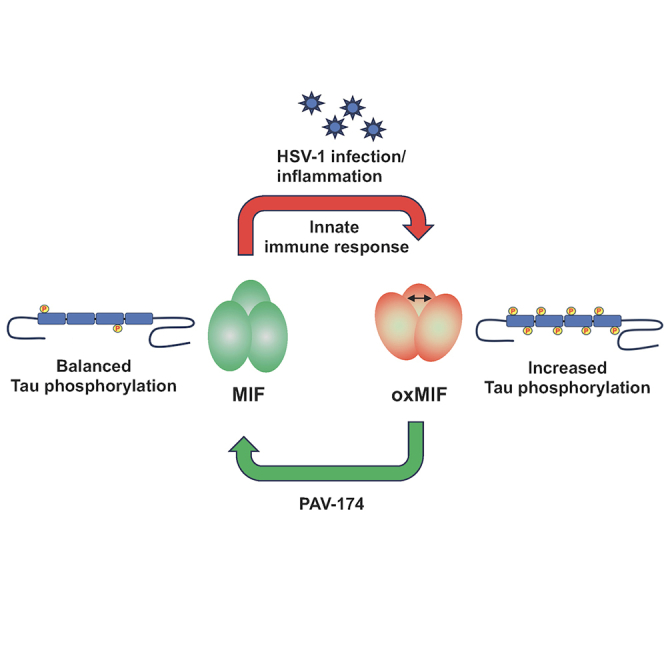

## Teaser

Müller-Schiffmann et al. identify oxidized MIF as a molecular hub relaying the effects of external stressors (e.g., infection with HSV-1) to cellular pathology associated with sporadic Alzheimer’s disease. Small-molecule compound PAV-174/ PAV-617 revert oxidized MIF and contingent tau phosphorylation and aggregation independent of viral infection *in vitro* and *in vivo*.

## Introduction

Despite an abundance of epidemiological evidence highlighting external risk factors critically contributing to the emergence of sporadic neurodegenerative diseases,^1^ the exact molecular mechanisms of how these factors are relayed to the well-characterized neuropathological or key cellular and molecular features of neurodegenerative diseases have remained unknown. Among those risk factors are conditions as diverse as obesity, diabetes, trauma, chronic infections, inflammation, or immune activation.^1^^,^^2^^,^^3^ Although known not to cause neurodegenerative diseases directly, the subchronic exposure to neurotropic viruses has been associated with the occurrence of, for example, Parkinson’s disease with influenza virus,^4^ dementias including Alzheimer’s disease (AD) with herpes viruses,^5^^,^^6^^,^^7^ or others.^8^ As possible mechanisms, either the molecular interaction between the virus and critical host cell factors or indirect effects of the virus through immune activation or its elicited cellular responses have been proposed.

During deep evolutionary time, in the arms race between viruses and their host cells, viruses have identified those host proteins that can be repurposed from their physiological functions, basically unveiling their “moonlighting” functions,^9^ for the virus’ maturation. Virus propagation occurs through a series of discrete steps, and the massive repurposing of distinct host proteins leads to a disruption of proteostasis, which eventually results in protein misfolding and, depending on the cellular context, protein aggregation.^10^ The identification of particular host proteins diverted by a virus and involved in balancing proteostasis in order to avoid protein aggregation is therefore also a strategy to identify pathophysiological mechanisms of protein aggregation in the absence of the virus. It is likely that the same host proteins balancing proteostasis are also disturbed by other external stressors or risk factors. These host proteins originally identified as host-viral molecular interfaces may thus have a more general significance beyond virus-specific effects, e.g., as general converging hubs or relays for external stressors.

The discovery of novel antiviral drugs has so far primarily focused on virus-encoded proteins, which makes intuitive sense, since the virus is the causative agent of virus-induced disease. The dependence of viral replication on host factors provides an alternative drug target, which is not limited to nucleic acid replication but also includes capsid assembly that can be reconstituted in cell-free protein synthesis and assembly (CFPSA) systems, enabling alternative drug screens.^11^^,^^12^^,^^13^^,^^14^ Drug targeting of cellular host factors has several advantages: first, drug resistance development is greatly diminished since the selection of host factors is on a much longer timescale than viral replication cycles. Second, the effects caused by host factor recruitment can give clues as to the basis for virus-induced cellular pathology, which ultimately causes clinical disease.^10^ Third, the ability to target a small subpopulation of specific host proteins recruited for their moonlighting functions might, in principle, not affect their canonical functions in cellular homeostasis. Bundling these advantages is challenging to obtain by rational design, but they can be gleaned from the viruses that achieved those innovations by natural selection.^10^

Herpes simplex virus (HSV-1) is a human pathogenic virus that in rare cases causes overt encephalitis but endemically stays latent in sensory neurons being reactivated upon a variety of conditions mainly related to immune challenges. Reactivation of latent herpes virus infection has been associated to AD even though the exact molecular mechanisms of this connection remain unknown.^6^^,^^15^^,^^16^^,^^17^ For example, a positive association of the presence of anti-HSV-1 antibodies to increased levels of phosphorylated tau in cerebrospinal fluid (CSF) of patients with AD has been demonstrated.^18^

We here present the discovery of a host protein-targeted antiviral drug (PAV-174) highly active against HSV-1 in human brain organoids and human neuronal cell lines with a clear structure-activity relationship (SAR). After target identification by drug resin-affinity chromatography (DRAC), we demonstrate by nuclear magnetic resonance (NMR) spectroscopy that the lead compound directly targets and partially reverses a distinct oxidized conformation of macrophage migration inhibitory factor (MIF). MIF is a homo-trimeric inflammatory cytokine with tautomerase, disulfide reductase, and likely other enzymatic activities. HSV-1 infection increases MIF levels,^19^ and MIF has been detected as part of virions of HSV-1 themselves.^20^ In addition, MIF plays a role in AD-related pathology, since MIF is associated with Aβ plaques^21^ and a deficiency of MIF decreases tau phosphorylation in a mouse model of AD.^22^ Increased levels of MIF have been reported in patients with AD, and CSF levels of MIF showed a robust correlation with phosphorylated tau.^23^ Therefore, MIF has recently been suggested as a potential biomarker for AD.^24^ As such, MIF has also been proposed to predict pre-symptomatic brain neurodegeneration in diabetic patients.^25^ Although it has been speculated that MIF has protective compensatory functions in AD,^26^ other reports showed that MIF may play a role in Aβ accumulation and Aβ-mediated toxicity.^21^ Interestingly, alternative oxidized conformers of MIF (oxMIF) have been detected in chronic inflammation including AD.^27^^,^^28^ However, the distinct role of oxMIF in the context of a potential biomarker or in AD-related molecular pathology has not been studied so far.

Within the work presented here, we validated MIF, especially its oxMIF conformation with the help of an original conformation-sensitive antibody, as a critical host factor for HSV-1 replication and demonstrated its role in tau molecular pathology relevant to neurodegenerative diseases such as AD or the frontotemporal dementias (FTDs). PAV-174 reduced HSV-1-induced tau phosphorylation via the Akt/GSK3β signaling pathway but was also active in the absence of infection *in vitro* and *in vivo*. OxMIF is capable of directly inducing tau phosphorylation, and we detected increased amounts of oxMIF in brain tissue of AD patients and after infection of differentiated neurons with HSV-1. Thus, oxMIF appears to be a missing molecular link connecting HSV-1 infection, and possibly other risk factors, with cellular pathology characteristic for neurodegenerative diseases involving aberrant tau phosphorylation or aggregation.

## Results

### PAV-174 prevents HSV-1 infection in differentiated LUHMES cells and human brain organoids

**Figure 1:**
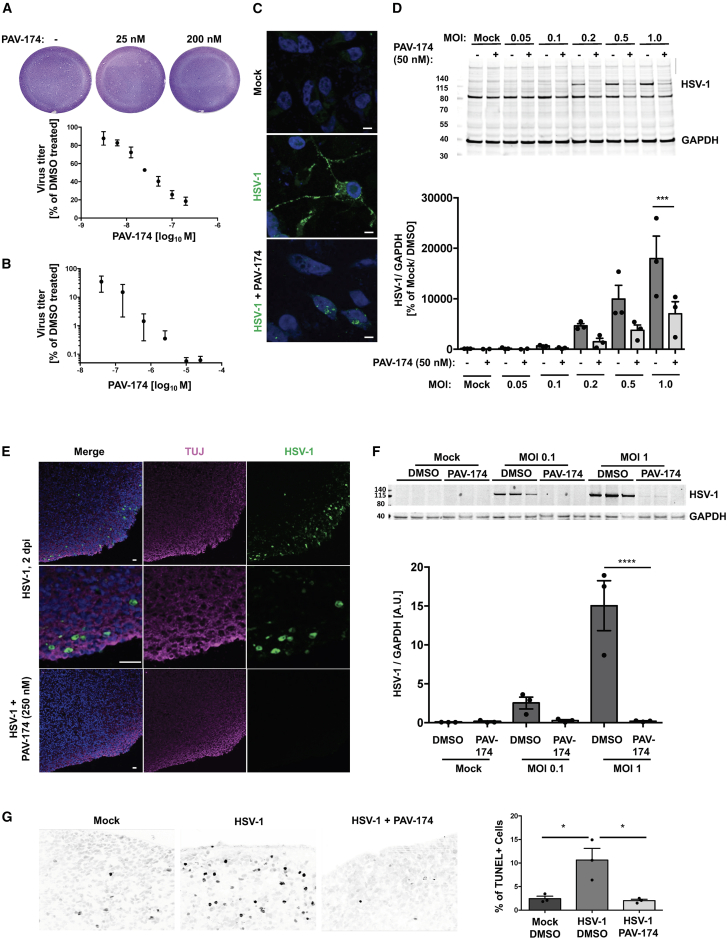
PAV-174 prevents HSV-1 infection in differentiated LUHMES and human brain organoids (A and B) PAV-174 antiviral activity was determined by plaque assay (A) or in-cell ELISA (B) in Vero cells infected with HSV-1. Each data point was normalized to the negative control and displays the mean ± SEM of three experiments (*n* = 3). IC_50_ of PAV-174: 34 nM (A) and 21 nM (B). (C) Immunocytochemistry of differentiated LUHMES cells infected with HSV-1 either treated with DMSO or PAV-174. Viral antigens (HSV-1-gC; green) present within the cytoplasm and axons of non-treated cells but not within the nuclei (DAPI) were reduced in the presence of PAV-174 (scale bars, 10 μm). (D) Western blot of cell lysates derived from differentiated LUHMES cells that were infected with HSV-1 and treated with DMSO or PAV-174. The upper signal represents an HSV-1 antigen detected with ab9533. GAPDH served as internal control. The diagram shows the normalized HSV-1 signals from three independent experiments (*n* = 3). Two-way ANOVA (Sidak’s post hoc). (E) Staining of human brain organoids infected with HSV-1 and treated with DMSO or PAV-174. HSV-1-gC (green) and TUJ-1-positive neurons (magenta) were detected in the outer layer of the organoids. Infection with HSV-1 was completely abolished when PAV-174 was present (scale bars, 30 μm). (F) Western blot with lysates from HSV-1-infected brain organoids. HSV-1 signals were normalized with GAPDH. The diagram represents the HSV-1 signals from three infected organoids per group (*n* = 3). Two-way ANOVA (Sidak’s post hoc). (G) PAV-174 lowered cell toxicity mediated by HSV-1 infection. TUNEL-positive cells within infected brain organoids were reduced by PAV-174. For each condition, three different organoids (*n* = 3) and at least 10 images of every organoid were analyzed. One-way ANOVA (Tukey’s post hoc). Data represent the mean ± SEM. ∗*p* < 0.05; ∗∗∗*p* < 0.001; ∗∗∗∗*p* < 0.0001. See also Figure S1.

From a CFPSA assay screen of a chemical library^12^^,^^29^ similar to what we have successfully described for rabies virus,^11^ human immunodeficiency virus,^13^ and respiratory viruses,^14^ an early lead compound (PAV-645) was identified with activity for inhibiting herpes virus capsid assembly by targeting an essential host protein. This compound was subsequently optimized for activity over seven generations of analog synthesis and screening, to an improved compound PAV-174 that inhibited HSV-1 replication in a dose-dependent manner in HSV-1-infected Vero cells, both in a standard plaque assay (Figure 1A) and in an in-cell ELISA (Figure 1B) with an IC_50_ of 34 or 21 nM, respectively, and an CC_50_ of 1.2 μM (Table 1; Figure S1B). Structural analogs displayed different antiviral potencies, indicating an SAR as evidence for specific activity (Table 1). In addition, the compound was also active in differentiated primary neuron-like human dopaminergic Lund human mesencephalic (LUHMES) cells (Figures 1C and 1D), as well as in differentiated human brain organoids (Figures 1E and 1F), where it also lowered HSV-1-induced neurotoxicity (Figure 1G). This indicated clear antiherpetic activity in several independent systems, including human primary-like cells.

**Table 1:** Structures of compounds including IC_50_ and CC_50_ values measured by in-cell ELISA for antiviral (HSV-1) activity and MTT assays, respectively

See also Figure S1.

### PAV-174 binds MIF at its multimerization interface

**Figure 2:**
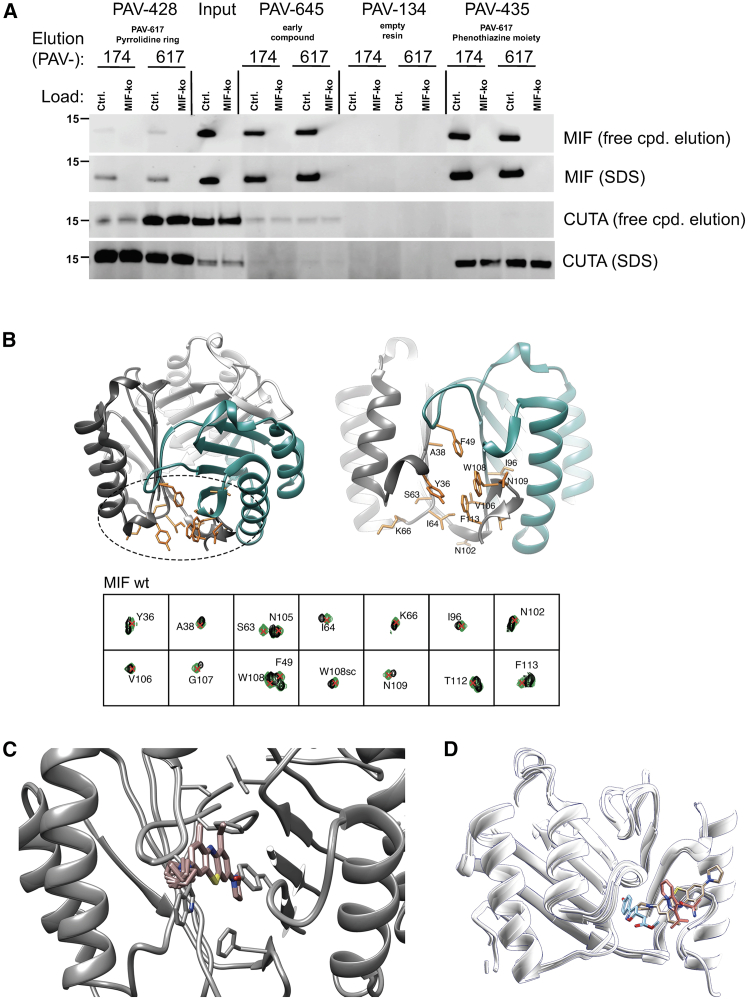
PAV-174 binds MIF at the multimerization interface (A) MIF and CUTA bind to PAV-617 resins at different moieties. PAV-617 was either coupled via its pyrrolidine ring (PAV-428) or its phenothiazine moiety (PAV-435). As controls, PAV-645 resin or empty resin (PAV-134) were used. Elution of bound proteins from SH-SY5Y-tau-P301S control and MIF-KO lysates with free compound and stripping of the resins with SDS revealed a different binding of MIF and CUTA to the resins. No MIF or CUTA signals were detected with PAV-134. (B) Structure of the MIF trimer interface with the residues showing compound PAV-174-induced CSP in the ^15^N-HSQC spectra (Figure S2D) represented in orange sticks. Each monomer is displayed in a different color. (C) Structure of the compound PAV-174 in complex with MIF calculated with the *N*MR^2^ protocol using ligand to protein’s aromatics and ligand to protein’s methyl distance restraints. The 10 best calculated poses have been retained, i.e., the poses with the lowest target function. (D) Overlay of binding poses of PAV-174 (beige, PDB ID: 8CA0), ISO-1 (blue, PDB ID: 1LJT), and AV1013 (orange, PDB ID: 3IJG) on MIF.^35^ See also Figure S2.

In order to determine the cellular target of PAV-174, we performed DRAC with the immobilized parent compound PAV-645. We used pig brain lysates (by availability) as material, and bound proteins were eluted with an excess of free PAV-645. Several proteins were identified by mass spectrometry, some of which have been described in the context of HSV-1- or AD-related research (Figure S2A): MIF^30^, cutA divalent cation tolerance homolog (CUTA^31^), glutathione-S-transferase Pi1 (GSTP1^32^), peroxiredoxin 2,^33^ and calmodulin-like 3.^34^ We validated these findings for MIF and CUTA by DRAC with SH-SY5Y neuroblastoma cells but failed to detect GSTP1 within the eluate (Figure S2B). To further characterize the binding of MIF and CUTA to PAV-174, we generated two additional resins presenting the close structural and functional homolog PAV-617 that was either coupled via its pyrrolidine ring or its phenothiazine moiety. Both, MIF and CUTA were eluted by free PAV-174 and PAV-617. However, MIF and CUTA showed a different preference to PAV-617 dependent on the coupling position (Figure 2A).

Even though the results suggested that multiple proteins, or a multiprotein complex, could be ligands of the active compound, we focused on MIF because of its known role in infection and neurodegenerative disorders.^30^^,^^36^^,^^37^^,^^38^ In order to confirm a direct binding of PAV-174 to MIF, we measured ^15^N-heteronuclear single quantum coherence (HSQC) spectra of PAV-174 with recombinant wild-type MIF (Figure S2C). Spectra revealed discrete binding sites (Figure S2D), suggesting specific binding. An NMR structure of recombinant MIF together with PAV-174 demonstrated a binding site at the interface of MIF monomers forming a trimer (Figures 2B and 2C). The MIF-PAV-174 complex structure revealed a ligand pose in the aromatic hinge formed by the residues Tyr36, Ile64, Val106, Trp108, and Phe113. PAV-174 is in particular inserting into the hinge by forming two π-π interactions, one with Trp108 from a monomeric subunit and the second with Tyr36 from a different monomeric subunit. The long tricyclic aromatic core moiety of PAV-174 engages in interactions with a more important number of residues than it is typically observed from other molecules known to bind at the same binding pocket, such as ISO-1 or AV1013.^35^ Indeed, ISO-1 inserts deeper into the binding pocket, at the location of the embedded pyrrolidine in beta of the ethyl aliphatic chain (Figure 2D). The ethyl aliphatic locates in the same region as the isopropyl chain of AV1013, suggesting an importance of a C2/C3 aliphatic substitution at this location to establish hydrophobic interactions. Finally, the part of PAV-174 that points out of the pocket is engaging in π-π interaction with Trp108, and this interaction seems to be missing for ISO-1 and AV1013. ISO-1 misses also the aliphatic C2/C3 chain that is observed to collocate for AV1013 and PAV-174.

### PAV-174 reduces tau phosphorylation also in the absence of infection in an MIF- and dose-dependent manner

**Figure 3:**
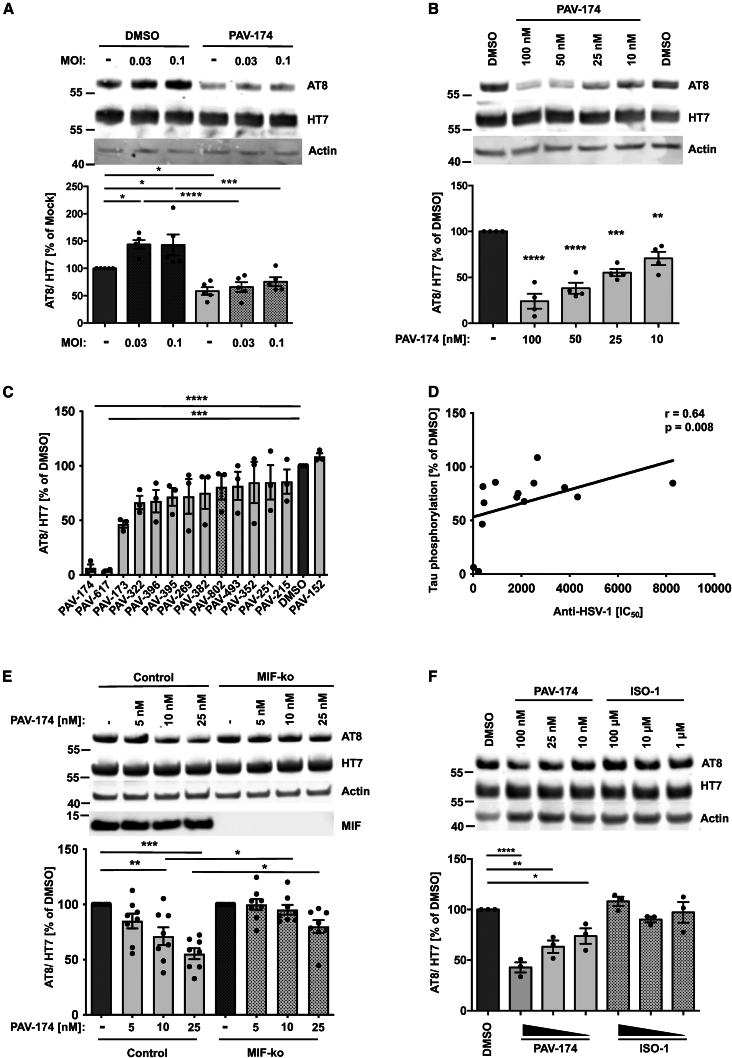
PAV-174 reduces tau phosphorylation also in the absence of infection in an MIF- and dose-dependent manner (A) Infection of SH-SY5Y-tau-P301S cells with HSV-1 increased tau phosphorylation (AT8). This increase was absent in the presence of PAV-174. The diagram shows the AT8 signals normalized to total tau (HT7) from five independent experiments (*n* = 5). Actin served as loading control. Two-way ANOVA (Sidak’s post hoc). (B) PAV-174 dose-dependently reduced tau phosphorylation also in the absence of viral infection. SH-SY5Y-tau-P301S cells were treated with PAV-174 that significantly reduced tau phosphorylation. The values from four independent experiments (*n* = 4) are presented. One-way ANOVA (Dunnett’s post hoc). (C) SAR of PAV-174 analogs on tau phosphorylation. SH-SY5Y-tau-P301S cells were treated with 500 nM of compounds. The diagram shows the normalized AT8 signals from three independent experiments (*n* = 3). One-way ANOVA (Dunnett’s post hoc). (D) The effects of the compounds on tau phosphorylation positively correlated to their capacity to inhibit HSV-1 replication. Spearman’s rho coefficient and *p* value are indicated. (E) Reduction of tau phosphorylation by PAV-174 is MIF dependent. SH-SY5Y-tau-P301S control and -MIF-knockdown cells were treated with indicated concentrations of PAV-174. A significant reduction of tau phosphorylation by PAV-174 was only observed in control but not in MIF-knockdown cells. The diagram shows the average of normalized AT8 values as percentage of DMSO-treated cells derived from eight independent experiments (*n* = 8). Two-way ANOVA (Sidak’s post hoc). (F) The MIF inhibitor ISO-1 does not modulate tau phosphorylation. SH-SY5Y-tau-P301S cells were treated with indicated amounts of PAV-174 or ISO-1. Up to 100 μM ISO-1 did not reduce tau phosphorylation in three independent experiments (*n* = 3). Data represent the mean ± SEM. One-way ANOVA (Dunnett’s post hoc). ∗*p* < 0.05; ∗∗*p* < 0.01; ∗∗∗*p* < 0.001; ∗∗∗∗*p* < 0.0001. See also Figures S3 and S4.

In order to analyze the effects of PAV-174 in a cellular model for neurodegenerative diseases, we generated a neuroblastoma-derived cell line stably overexpressing human tau (2N4R), including the familial mutation P301S (SH-SY5Y-tau-P301S). These cells could efficiently be infected with HSV-1 (Figure S3A). HSV-1 infection increased tau phosphorylation at residues Ser202 and Thr205 (AT8) in SH-SY5Y-tau-P301S cells (Figure 3A). Compound PAV-174 reduced tau phosphorylation at these sites but also in the absence of virus in a dose-dependent manner at low nanomolar concentrations (Figure 3B). A dose-dependent reduction of phosphorylation was also detected at Thr231 (AT180) and only at higher concentrations on Thr181 (AT270), indicating a specific effect (Figures S3B and S3C). The tau phosphorylation-inhibiting effects displayed an SAR (Figures 3C and S3D) that positively correlated with the anti-HSV-1 activity (Figure 3D). The effect of PAV-174 on inhibiting tau phosphorylation was MIF dependent. This was demonstrated in SH-SY5Y-tau-P301S cells that were knocked down for MIF (Figure S3E). PAV-174 effects on tau phosphorylation were absent in MIF-knockdown compared to the control cells and cells where CUTA have been knocked down (Figures 3E and S3F), emphasizing the role of MIF over CUTA. Of note, PAV-174 did not act like other well-studied MIF inhibitors such as ISO-1,^21^^,^^22^ which did not modulate tau phosphorylation even at high micromolar concentrations (Figure 3F). The absence of an ISO-1 effect indicates that the tautomerase activity is not important for MIF’s regulation of tau phosphorylation. Nor did PAV-174 interact with MIF’s close relative, D-dopachrome tautomerase (D-DT; Figure S3G). MIF modulates a range of different interconnected signaling cascades, including ERK1/2 MAP, phosphatidylinositol kinase-3 (PI3K)/Akt, and JNK, via binding to the chemokine receptors CXCR2, CXCR4, and CD74.^39^^,^^40^^,^^41^ PAV-174 increased the phosphorylation of Akt at Ser473 as well as GSK3β at Ser9 in a dose-dependent and MIF-dependent manner, since the effects were significantly less pronounced in SH-tau-P301S-MIF-knockdown cells (Figures S4A and S4B). We conclude that the effects of PAV-174 on MIF-dependent tau phosphorylation are executed via Akt and GSK3β.

Of note, HSV-1 capsid antigens accumulated faster in the MIF-knockdown cell line with increasing multiplicity of infection (MOI). This effect was most pronounced at an MOI of 0.2 (Figure S4C), whereas infectivity was not changed (Figure S4D), thereby increasing the HSV-1 antigen/titer ratio and corroborating our hypothesis that HSV-1 capsid assembly inhibition leads to an accumulation of non-assembled viral proteins. Full activity of lead compound PAV-174 was dependent on the presence of MIF. In SH-SY5Y-tau-P301S-MIF-knockdown cells, PAV-174 did not exert comparable titer-lowering effects (Figure S4E), indicating that MIF or a subset of MIF conformers (see the following paragraph), likely mediates the antiherpetic effects of PAV-174.

### PAV-174 decreases tau phosphorylation and aggregation *in vivo*

**Figure 4:**
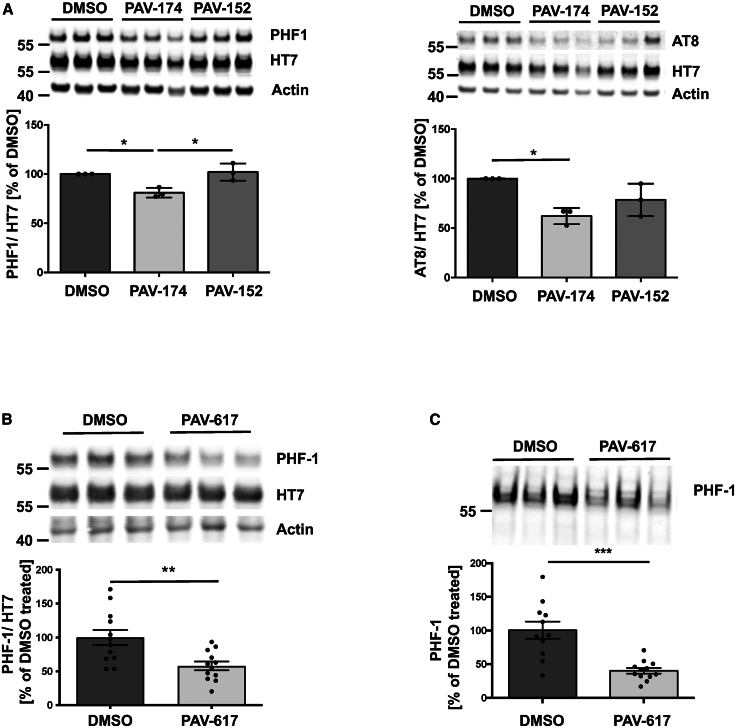
PAV-174 decreases tau phosphorylation and aggregation *in vivo* (A) PAV-174 but not the non-functional analog PAV-152 reduced tau phosphorylation (left: PHF-1; right: AT8) in differentiated neurons derived from human iPSCs. The diagram shows the average values of three independent experiments each with three technical replicates (*n* = 3). One-way ANOVA (Tukey’s post hoc). (B) Reduction of tau phosphorylation was observed *in vivo* after treating tau58/2 mice with 5 mg/kg of PAV-617. HT7-normalized phosphorylated tau (PHF-1) was reduced in the homogenates of the compound-treated mice. The diagram shows the average signals of 12 mice per treatment group derived from three independent western blots. Unpaired two-tailed *t* test. (C) Significantly less phosphorylated tau (PHF-1) was detected in the sarkosyl-insoluble fraction of PAV-617-treated mice. The diagram shows the average signals of 12 mice per treatment group derived from two independent western blots. Data represent the mean ± SEM. Unpaired two-tailed *t* test. ∗*p* < 0.05; ∗∗*p* < 0.01; ∗∗∗*p* < 0.001. See also Figure S5.

To corroborate the effects of PAV-174 on tau phosphorylation in primary-like human cells, we treated cortical neurons differentiated from human iPSCs of a patient carrying the MAPT IVS10 + 16 mutation (V97).^42^ We observed reduced phosphorylation at position Ser396 and Ser404 of tau that are also targeted by GSK3β,^43^ detected by the specific antibody PHF-1, and at the AT8 site (Figure 4A). In order to validate the effects of PAV-174 *in vivo*, we treated 2-month-old male tgTau58/2 mice^44^ (overexpressing human tau with the P301S mutation) with the structural analog PAV-617 (see Table 1) three times weekly for 4 weeks. We chose PAV-617 since it was less toxic in mice and showed a substantially higher bioavailability likely due to lower protein binding of 91.2% compared to 99.9% for PAV-174 (Figure S5A). Both compounds had comparable activity regarding MIF binding (Figure 2A) and inhibition of tau phosphorylation (Figures 3C, 3D, and S5B). PAV-617 showed high bioavailability following intraperitoneal administration and good tissue penetration, including the brain (Figures S5C and S5D). We observed significantly reduced tau phosphorylation (PHF-1 and AT8) in brain homogenates of PAV-617-treated mice (Figures 4B and S5E). Furthermore, sarkosyl-insoluble tau species were significantly less abundant in the brain tissue of treated mice (Figures 4C and S5F), indicating less tau aggregation as well. Together, these observations confirm the potency of these compounds in reducing tau phosphorylation and aggregation in disease-relevant models *in vitro* and *in vivo*.

### The oxMIF conformer is elevated in postmortem brains of patients with AD

**Figure 5:**
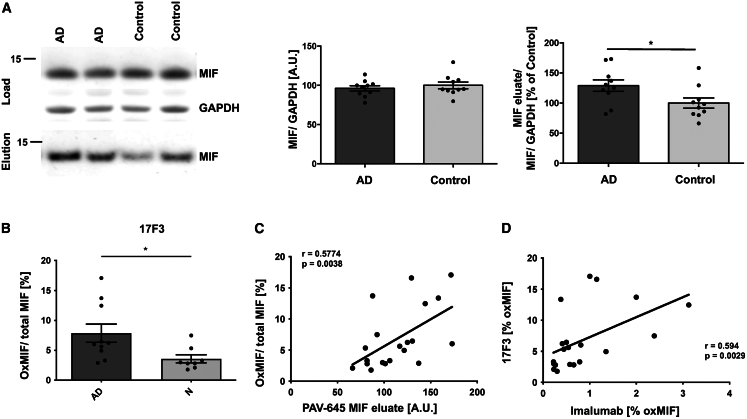
The oxMIF conformer is elevated in postmortem brains of patients with AD (A) DRAC analysis of 20 brain samples from patients with AD and healthy controls. Left: western blot (representative section); upper: the loading control (MIF and GAPDH) from brain homogenates applied to PAV-645 resin; and lower: the pulled-down and PAV-174-eluted MIF. The GAPDH-normalized MIF protein levels within the brain samples did not significantly differ between AD samples and controls (left diagram). The eluted MIF was normalized to the input MIF/GAPDH signal (right diagram). Significantly more MIF was precipitated from AD brain samples by DRAC (displayed as percentage of the average in control samples). Each data point represents the average of two independent experiments. Unpaired two-tailed *t* test. (B) Increased oxMIF/total MIF in AD brain samples was detected with 17F3 by sandwich ELISA. Each data point represents the average of two independent brain sample preparations. Outliers were excluded using the ROUT method (Q = 5%). Unpaired two-tailed *t* test. (C) The oxMIF levels detected by 17F3 positively correlated with the amounts of MIF species eluted from PAV-645 resin. Spearman’s rho coefficient and *p* value (one-tailed) are indicated. (D) The oxMIF levels detected by 17F3 positively correlated with those determined by imalumab. Spearman’s rho coefficient and *p* value (one-tailed) are indicated in the graph. Data represent the mean ± SEM. ∗*p* < 0.05. See also Figure S6.

To investigate a role of MIF in the brains of patients with AD, we obtained brain tissue from the middle frontal gyrus derived from patients with AD or healthy controls (Figure S6A). Braak and Thal staging of *postmortem* brains had been carefully conducted and was significantly higher in the AD group, whereas age and *postmortem* delay was equal (Figure S6B). When we performed DRAC with the original compound PAV-645 and eluted with the more efficient compound PAV-174, we observed a higher concentration of MIF derived from brain homogenates of patients with AD compared to the healthy controls (Figures 5A, S6C, and S6D).

MIF has been reported in at least two conformers, an oxidized and a reduced conformer. A commercially available conformation-sensitive antibody for oxMIF, termed imalumab, has been generated,^45^ which we used to establish a sandwich ELISA allowing the detection of oxMIF in biological samples. As positive controls, we used wild-type MIF treated with Proclin300^28^ and MIF-C80W, both of which mimic the oxMIF conformation.^46^ Total MIF levels were determined by western blot, since in ELISA, seemingly all commercial MIF antibodies had a bias toward oxMIF (data not shown). In these quantifications, the oxMIF/total MIF ratio was higher in brains from patients with AD (Figure S6E), and the levels of oxMIF positively correlated with the MIF species eluted by DRAC (Figure S6F).

In order to independently validate these results with another oxMIF-specific antibody, we generated our own monoclonal antibody (mAB) against an oxMIF epitope using recombinant MIF-C80W as an immunogen.^47^ We identified mAB 17F3 that recognizes a linear epitope within MIF (^45^DQLMAF^50^), non-overlapping with that of imalumab (Figure S6G). 17F3 binds to H_2_O_2_- and Proclin300-induced oxMIF but not to native MIF (Figure S6H). Similar to imalumab, 17F3 detected more oxMIF in brain tissue of patients with AD (Figure 5B), correlating with the MIF species eluted by DRAC (Figure 5C). In addition, the oxMIF levels detected by 17F3 also correlated with those determined with imalumab (Figure 5D). Importantly, the H_2_O_2_-induced oxMIF, that we used for defining oxMIF, for screening the antibody, and as a standard for ELISA, maintained a native-like trimeric conformation as it was still detectable by yet another anti-MIF antibody, termed 57D9, generated in a similar screen and that was conformation-specific to the trimeric conformation of MIF^48^ (Figure S6I). Of note, we also detected an increase of oxMIF species in the supernatants of HSV-1-infected differentiated LUHMES cells (Figure S6J) but found no changes in total MIF (Figure S6K), corroborating the role of oxMIF in disease-associated states.

### OxMIF is a direct driver of tau phosphorylation modulated by PAV-174

**Figure 6:**
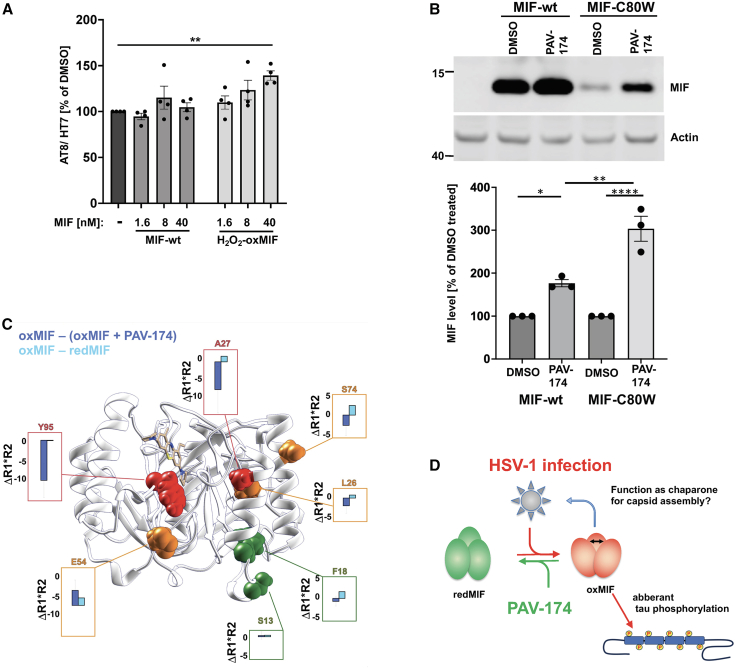
OxMIF is a direct driver of tau phosphorylation modulated by PAV-174 (A) Exogenous applied oxMIF enhances tau phosphorylation. SH-SY5Y-tau-P301S control cells were treated with recombinant wt-MIF or H_2_O_2_-oxMIF for 6 h. OxMIF but not non-oxidized wt-MIF dose dependently induced tau phosphorylation in four independent experiments (*n* = 4). One-way ANOVA (Dunnett’s post hoc). (B) Transiently expressed recombinant MIF-C80W is stabilized within HEK-MIF-knockdown cells by PAV-174. HEK-MIF-KO cells (left lane) were transfected with pLNCX-MIF-wt-rescue or pLNCX-MIF-C80W-rescue and treated with either PAV-174 or DMSO as control. PAV-174 led to a significant stabilization of MIF-wt and MIF-C80W. The relative stabilization of MIF was stronger for MIF-C80W. The diagram shows the average values of three independent experiments (*n* = 3). One-way ANOVA (Tukey’s post hoc). (C) Structural dynamic cancellation of compound PAV-174 on oxMIF visualized on the structure of MIF bound to PAV-174. For the residues highlighted as spheres, the ^15^N NMR relaxation product difference (ΔR1∗R2) between oxMIF and oxMIF + PAV-174 (deep blue) and between oxMIF and redMIF (cyan) is displayed in the boxes. The residues colored in red show a strong cancellation of the motion on the microsecond timescale upon addition of PAV-174, moderate in orange, and little or no effect in green. (D) Hypothetic model of MIF isoforms implicated in HSV-1 life cycle and tau phosphorylation. Data represent the mean ± SEM. ∗*p* < 0.05; ∗∗*p* < 0.01; ∗∗∗∗*p* < 0.0001. See also Figure S7.

To analyze the relevance of oxMIF on tau phosphorylation, we exogenously applied recombinantly expressed wild-type MIF, H_2_O_2_-induced oxMIF, or MIF-C80W^46^ and administered these proteins at defined dosages to SH-SY5Y-tau-P301S cells. We observed a weak but significant induction of tau phosphorylation by H_2_O_2_-induced oxMIF as well as MIF-C80W but not with wild-type MIF after 6 h (Figures 6A, S7A, and S7B). This effect was not observed in SH-SY5Y-tau-P301S-MIF-knockdown cells (Figure S7B). By using DRAC, we observed stronger binding of H_2_O_2_-oxMIF to PAV-645 compared to wild-type MIF (Figure S7C).^49^ In a first attempt to transiently express MIF-C80W on an MIF-knockdown background in HEK cells for the analysis of oxMIF-specific functions, we recognized that MIF-C80W expression was much weaker than that of wild-type MIF (Figure S7D). However, as predicted from our structural studies (Figure 2), MIF and especially MIF-C80W were stabilized when the cells were treated continuously with PAV-174 during the transient expression of MIF (Figure 6B), indicating that PAV-174 converts the inherently unstable oxMIF to redMIF, which is thereby stabilized. This prompted us to analyze the binding of PAV-174 to oxMIF by NMR (Figure 6C). The ^15^N-HSQC spectra of wild-type MIF (redMIF) and H_2_O_2_-treated wild-type MIF (oxMIF), in the presence or absence of PAV-174, revealed chemical shift perturbations (CSPs) upon PAV-174 addition, confirming that PAV-174 bound to both MIF species. However, the low alignment between the chemical shifts of oxMIF and redMIF did not allow to transfer the assignments of the redMIF- onto the oxMIF-^15^N-HSQC spectrum. Therefore, the binding site of PAV-174 in oxMIF could not be definitely determined to be precisely the same as for wild-type redMIF. It is worth noticing though that the resonance and shifts corresponding to the Trp108 side chain are easily aligned between MIF conformers, suggesting that the PAV-174 binds at the same site or in close proximity in both conformers.

To investigate a potential effect of PAV-174 on differential multimerization of wild-type MIF and MIF-C80W, we performed high-pressure liquid chromatography-multi-angle laser light scattering (HPLC-MALS), using a size exclusion column. The high-pressure liquid chromatography runs were performed for wild-type and the C80W mutant in the presence or absence of PAV-174 (Figure S7E). The addition of PAV-174 did not lead to different retention times in both MIF species. Furthermore, the molecular weight of the observed species was calculated for each sample: wt-MIF, 34,140 kDa ±2%; wt-MIF + PAV-174, 35,270 kDa ±2%; MIF-C80W, 37,890 kDa ±7%; and MIF-C80W + PAV-174, 37,180 kDa ±5%, using the Astra software, correcting the Rayleigh ratio by the UV absorbance at 280 nm. These results indicated that MIF is present as a trimer regardless of the interaction with PAV-174 and that PAV-174 does not perturb the monomer-trimer equilibrium or the oligomerization of both wt-MIF and MIF-C80W (Figure S7E). The different retention times of wild-type MIF and MIF-C80W are likely due to different conformations of both MIF species that have been described.^46^ The resolution of the HPLC-MALS is not in the atomic range and therefore only reports conformation changes at the quaternary structure scale (i.e., the formation of multimers) and, to some extent, the tertiary structure scale (i.e., unfolding). For this reason, it cannot be excluded that PAV-174 affects the secondary/tertiary structure or dynamics for the two MIF conformers.

## Discussion

The molecular interfaces linking external risk factors to the development of neurodegeneration are poorly understood. Here, we demonstrate that oxMIF is a cellular response to an external stressor, HSV-1, that mediates tau phosphorylation, a critical event in the genesis of neurodegenerative diseases such as AD or subtypes of FTDs. We also identified a compound that, upon binding MIF, stabilizes a non-oxMIF conformation and thereby reverses tau phosphorylation and aggregation *in vitro* and *in vivo*, respectively, demonstrating that this class of pharmaceuticals could become relevant for treating sporadic forms of neurodegenerative diseases such as sporadic AD.

Independent of the link to tau phosphorylation, blocking oxMIF by PAV-174 efficiently interfered with HSV-1 replication at low nanomolar concentrations—acyclovir, the standard competitive inhibitor of herpes virus DNA polymerase, is efficient at only 8.5 μM IC_50_ in Vero cells.^50^ Remarkably, PAV-174 showed no induction of apoptosis in human brain organoids, indicating that it prevents HSV-1 infection without being neurotoxic under the conditions used. Since generalized herpes virus encephalitis remains a serious neurological diagnosis with a high mortality (20% even under acyclovir therapy) and severe lasting post-infection symptoms,^51^ the pharmacological target oxMIF and the class of compounds presented here may prove to significantly increase antiherpetic pharmacotherapy for the benefit of subacutely HSV-1-infected patients. Antiviral therapy targeting a host-viral interface is thus highly efficient and in continuity with our previous research on host-targeting antiviral compounds.^11^^,^^13^^,^^14^ PAV-174 and PAV-617 have previously been observed to have antiviral effects also on pox viruses.^52^ The relationship between these effects and the mechanisms discussed here are currently unknown but are worth to be investigated.

Intriguingly, despite the fact that inhibition of oxMIF by PAV-174 is pharmacologically highly effective, the presence of MIF appears not to be essential for the HSV-1 life cycle. The significant increase in level of capsid protein without the enhancement of viral titer argues that, as predicted, misassembly of HSV-1 capsid was achieved by either treatment with PAV-174 or knockdown of MIF but not to the degree of reducing infectious particles. The lack of a higher effect on reducing the viral titer could be explained by the presence of D-DT, a structural cellular homolog of MIF^53^ that has been reported to share essential functions with MIF.^54^ We did not consider engineering an additional knockdown of D-DT since we did not detect D-DT in DRAC, and the combined loss of MIF and D-DT has been shown to increase programmed cell death by inhibition of p53.^55^

Protein misfolding is ultimately the result of disturbed proteostasis, and, accordingly, protein misfolding diseases (PMDs), characterized by the deposition of misfolded, aggregated proteins, are fundamentally caused by disease-specific disturbed proteostasis. For the genetic cases of PMDs, it is easy to understand that a mutant protein is eventually more prone to misfolding or aggregation. It is less obvious how a similar misfolding is triggered by external factors in the majority of sporadic cases of PMDs. We have previously suggested that if a specific viral infection results in a cellular pathology similar to that characteristic for a given PMD, the dissection of host proteins that this virus recruits, e.g., for its (capsid) assembly, and that hence are causing the disruption in cellular proteostasis may reveal important clues about molecular interfaces involved in triggering the given PMD even in the *absence* of virus.^10^

We here demonstrate the usefulness of this concept: HSV-1 infection increases tau phosphorylation, a hallmark of aggregated tau, at PMD-relevant residues^43^ *in vitro*, and this can be prevented by PAV-174 in an MIF-dependent manner. Our finding that, even in non-infected cells, clear PAV-174 effects were seen can be taken as evidence that low levels of oxMIF, as detected in the brains of healthy control patients, are always present, which are, however, inherently unstable but activate signaling ending in tau phosphorylation.

Our data are in accordance with previous findings demonstrating that the absence of MIF decreased tau phosphorylation in a mouse model of AD.^22^ By binding to its canonical receptors, MIF interferes with Akt/GSK3β signaling that are important signaling cascades involved in tau phosphorylation,^41^ consistent with our observations that PAV-174 specifically reduces phosphorylation at sites modulated by GSK3β (AT8, AT180, and PHF-1). Accordingly, we did not observe a comparable effect of PAV-174 on preventing phosphorylation of residues not affected by GSK3β. Interestingly, a recent study reported that HSV-1 induced tau phosphorylation via TBK1,^56^ a pathway that is also affected by GSK3β upon viral infection.^57^ The authors suggested that phosphorylated tau could limit viral infection and have a neuroprotective effect. Along these lines, it has also been stated that HSV-1 infection can induce Aβ aggregation, serving an antiviral function.^17^ MIF has been coprecipitated with Aβ^58^ and been shown to associate with Aβ plaques.^21^ An implication of MIF in Aβ pathology is currently discussed controversially^30^ and also includes a potential beneficial function of MIF as a compensation for cognitive decline in AD.^26^

MIF is a key factor in mediating inflammatory responses and is also involved in relaying other external triggers of sporadic AD. For example, serum levels of MIF are markedly increased in patients suffering from insulin resistance and type 2 diabetes (summarized in Morrison and Kleemann^59^). The role of different MIF conformers is not yet widely discussed for differential functions in regulating physiological circuitry. Studies have reported increased oxMIF levels in chronic inflammatory diseases,^30^ of which some have been reported to present a higher risk to develop dementia, such as asthma, sepsis, psoriasis, ulcerative colitis, Crohn’s disease, and systemic lupus erythematosus.^28^^,^^60^^,^^61^^,^^62^^,^^63^^,^^64^ We thus speculate that oxMIF has the potential to be a molecular interface in relaying external events at a broader scale to tau phosphorylation and aggregation, thereby accelerating the risk to develop dementia. Along these lines, the increased oxMIF levels in our AD brain samples were likely induced by various external stressors and not restricted to HSV-1.

H_2_O_2_-induced oxMIF and MIF-C80W were sufficient to induce tau phosphorylation *in vitro*. However, endogenous MIF is necessary for this effect since increased tau phosphorylation was absent in MIF-KO cells. One explanation is that recombinant MIF has been shown to markedly induce the secretion of intracellular MIF constituting an autocrine loop.^65^ Therefore, oxMIF could be a potent stimulator of enhanced MIF secretion.

Since antibodies against β-sheeted components of MIF exhibited the largest anti-inflammatory potential,^45^ selective antibodies against oxMIF were generated and characterized.^28^^,^^46^ A phase 1 study of one of these antibodies, imalumab, in patients with solid tumors has been published.^66^ However, in the absence of follow-up publications, earlier clinical studies on imalumab in antitumor therapy seem to have been abandoned.^67^ Imalumab was developed from a native human IgG library, and its epitope was suggested to be exposed only in oxMIF.^28^^,^^45^^,^^46^ We decided to independently validate the results obtained with imalumab with our own oxMIF-specific antibody 17F3 raised against recombinant MIF-C80W. 17F3 displayed striking similarities to imalumab regarding selectivity for oxMIF. Although, 17F3, like imalumab, recognizes a linear and not a conformational (i.e., folding sensitive) epitope, the identified binding site does not overlap with that of imalumab and is of particular interest. It encompasses the β3 strand, including a hydrophobic pocket that is important for structural stability^68^ and is part of a conformational epitope mediating binding of MIF to CXCR2.^68^ Thus, oxidation of MIF, as well as the C80W mutation, could therefore modulate binding to CXCR2 and thereby induce a distinct function of oxMIF. A direct implication of CXCR2 in the PI3K/Akt/Gsk3β signaling pathway and in tau phosphorylation has been described.^69^^,^^70^

Recently, a structure of H_2_O_2_-oxMIF was published.^49^ Remarkably, our structural investigations reveal that binding of PAV-174 to oxMIF restores, to some degree, the redMIF conformation and thus may revert its proinflammatory effects, explaining its modulation on Akt signaling and tau cellular pathology. The effect of PAV-174 seems to be allosteric since the ^15^N-HSQC reveals remote CSPs from the binding site of PAV-174. Among the strongest CSPs, we observe Lys66, Ser63, Gln102, Ala38, and Ile96, which are all distant from the binding site of PAV-174. The differences in the spin relaxation rates ΔR_1_R_2_ were obtained by NMR^71^ and reveal an important reversal of the motion in the microsecond scale for the residues Ala27, Leu26, and Ser74, forming a network connecting the top-end of the two alpha helices. Furthermore, we can observe a regiospecificity of the effect of PAV-174 binding, as the residues Ser13 and Phe18, located at the bottom-end of the first alpha helix, do not see their dynamics affected upon PAV-174 binding as observed in the spin relaxation rate differences ΔR_1_R_2_.

The long-known epidemiological association between latent herpes virus reactivation and AD^3^^,^^16^^,^^72^^,^^73^^,^^74^ is opposed by much fewer studies unable to find this association in different cohorts.^75^^,^^76^ The molecular mechanism of potentially triggering effects of herpes virus activation have remained unexplained, even though several molecular scenarios have been proposed such as an interference of herpes virus with autophagy.^77^^,^^78^ Our results suggest that a small subfraction of MIF, present in the oxMIF conformation, presents the missing molecular link between HSV-1 infection and AD-like cellular pathology, since it has a role in both HSV-1 life cycle and tau phosphorylation.

Methylene blue (MB; PAV-802) is a phenothiazine core derivative, as are both PAV-174 and PAV-617. MB was previously reported to be an inhibitor of tau aggregation, including *in vivo*,^79^ and is currently studied in a clinical trial for the treatment of AD.^80^ For two independent reasons, we believe the activity observed in our assays is different from those observed for MB by others. First, in our *in vitro* assay, PAV-174 was around 2-logs more potent than PAV-802 in inhibiting HSV-1 replication or tau phosphorylation. PAV-617 retained that enhanced activity. Second, our NMR data suggest that the mode of action is likely different from that of MB since one of the unique pyrrolidine rings of PAV-174 (not present in MB) is deeply embedded in the hydrophobic pocket formed by Tyr36, Ile64, Val106, Trp108, and Phe113. We hypothesize that the strong hydrophobic interactions with residues located close to the core of the protein engages the allosteric network. The second pyrrolidine ring is pointing outward from the binding site of MIF. Despite its solvent exposure, its proximity with the indole of Trp108 suggests potential **π** interaction with this residue, which is facing the other side of Cys80, again suggesting its key importance in allosteric effects of PAV-174.

We put forward a model (Figure 6D) where we speculate that, upon HSV-1 infection, the induced oxMIF could function as a chaperone, or in some other role, in the process of HSV-1 capsid assembly. This general function of MIF has been described for SOD1, although the specific participation of the oxMIF conformer in the MIF-SOD1 interaction was not studied.^81^ In addition to the likely many specific functions of oxMIF, the presence of oxMIF increases tau phosphorylation. Both effects can be reverted by PAV-174 by re-establishing the redMIF conformation, thereby reducing both efficient viral maturation as well as aberrant tau phosphorylation. Our discovery of one particular conformer of MIF, oxMIF, which has been described as proinflammatory and disease associated in various contexts,^28^ and is upregulated in *postmortem* brains from patients with AD, now defines the known role of MIF in AD^21^^,^^30^ more precisely and corroborates a proinflammatory state in AD.

In conclusion, we have presented an oxMIF-targeting anti-HSV-1 compound class with several notable features related to neurodegenerative disease therapeutics. These compounds prevent aberrant tau phosphorylation and aggregation and hence are likely relevant to potential drugs against tau-dependent neurodegenerative disorders like AD, independent of HSV-1 infection. MIF, and possibly other proteins in conjunction with MIF in a multiprotein complex, are at the intersection between HSV-1 replication and AD-like cellular pathology providing a molecular basis for further analyzing the long-known epidemiological connection between these disorders. The reported functional pleiotropy of MIF suggests that also other AD risk factors may relay via the MIF molecular interface. Future studies will show the capability of mAb 17F3 to sensitively detect oxMIF in patient samples to better stratify at-risk patients and facilitate early, personalized intervention therapies in AD and other tauopathies.

### Limitations of the study

The oxMIF conformation of MIF is not well defined, and several post-translational modifications of MIF have been described as oxMIF (reviewed in Schindler et al.^82^). We chose the C80W mutation and H_2_O_2_-induced oxMIF as models. MIF-C80W has been demonstrated to mimic an oxMIF conformation that is recognized by imalumab,^46^ and the H_2_O_2_-induced conformation of wild-type MIF likely resembles the situation of MIF in an oxidizing environment.^49^ However, since the exact nature of physiological oxMIF is not resolved, their specific relevance for the disease-associated oxMIF conformation can only be indirect.

We cannot fully exclude off-target effects of PAV-174 and PAV-617 besides their effects executed via MIF. In addition to MIF, other factors co-eluted with MIF in our DRAC assay. Although CUTA did not show any relevance for the inhibition of tau phosphorylation by PAV-174 and GSTP1 could not be validated as target within SH-SY5Y cells, other proteins that were detected in the initial DRAC assay and that have not been further analyzed in this study could well be targeted by PAV-174, especially at higher concentrations, and therefore contribute to the effects.

The effects of PAV-617 *in vivo* were obtained in male tgTau58/2 mice; the described changes in tau neuropathology between 2 and 3 months are minimal,^44^ which is why we believe that our results are nevertheless representative. A meaningful statistical calculation of sex-specific effects regarding the presence of oxMIF in the brains of patients with AD (Figure 5) was not possible due to the small sample size for each sex.

## Resource availability

### Lead contact

Further information and requests for resources and reagents should be directed to the lead contact, Carsten Korth (ckorth@hhu.de).

### Materials availability

All unique/stable reagents generated in this study are available from the lead contact with a completed materials transfer agreement.

### Data and code availability

• The structure of MIF in complex with PAV-174 has been deposited in the Worldwide Protein Data Bank as PDB: 8CA0 (wwPDB deposition ID: D_1292128100) and is publicly available. Proteomics data from DRAC elution leading to MIF identification have been deposited in MassIVE as MassIVE:MSV000099711 (DOI: https://doi.org/10.25345/C56M33H2N). All data are available upon request.
• This paper does not report custom code.
• Any additional information required to reanalyze the data reported in this paper is available from the lead contact upon request.

## Acknowledgments

We thank Jesus Requena for critical discussions and Bruce C. Onisko, Christopher J. Silva, and Melissa L. Erickson Beltran for technical assistance. This research was funded by 10.13039/501100002347BMBF REMOVAGE (#01GQ1422A), the Research Commission of the Medical Faculty of the 10.13039/501100003484Heinrich Heine University Düsseldorf (#9772726), the DFG (KO1679/10-1,15-1), the 10.13039/501100008747Deutsche Alzheimer Gesellschaft, and a grant from Prosetta Biosciences.

## Author contributions

A.M.-S., C.K., and V.R.L.: conceptualization, writing – original draft, writing – review and editing; A.M.-S., S.K.T., J.O., A.H., I.P., V.B., A.A., S.W., S.Y., D.D., S.M., D.P., and D.S.: investigation; F.T.: investigation and writing – original draft; A.K., A. Rozemuller, A. Ramani, and J.G.: resources; V.R.L.: methodology; R.R.: methodology, supervision, and writing – original draft.

## Declaration of interests

V.R.L. is CEO, founder, and shareholder of Prosetta Biosciences. D.P. is CEO of Prosetta Bioconformatics Pvt. Ltd., a wholly owned Prosetta subsidiary. A.K., S.Y., D.D., S.M., and D.S. are full-time employees of Prosetta Biosciences. Patents have been filed on small-molecule compounds named in this paper by Prosetta Biosciences and on mAB 17F3 by C.K. and A.M.-S. The present research was, in part, supported by a grant from Prosetta Biosciences to C.K.

## STAR★Methods

### Key resources table

**Table:** 

REAGENT or RESOURCE	SOURCE	IDENTIFIER
**Antibodies**		

Anti-HSV-1	Abcam	ab9533; RRID:AB_307320
Anti-HSV-1 gC	Abcam	ab6509; RRID:AB_305531
Anti-HSV-1 VP5	Abcam	ab6508; RRID:AB_305530
Anti-GAPDH [A-3]	Santa Cruz Biotech.	sc137179; RRID:AB_2232048
Anti-TUJ-1	Sigma-Aldrich	T2200; RRID:AB_262133
Anti-MIF (polyclonal)	Sigma-Aldrich	HPA 003868; RRID:AB_1079290
Anti-MIF [10C3]	Biolegend	525501; RRID:AB_2563133
Anti-MIF [57D9]	This paper	N/A
Anti-oxMIF [Imalumab]	Creative Biolabs	TAB-H41; RRID:AB_2911180
Anti-oxMIF [17F3]	This paper	N/A
Anti-CUTA (polyclonal)	Sigma-Aldrich	HPA 064369; RRID:AB_2685248
Anti-GSTP1	Merck-Millipore	ABS1650
Anti-tau (total) [HT7]	Thermo Fisher Scientific	MN1000; RRID:AB_2314654
Anti-phospho-tau [AT8]	Thermo Fisher Scientific	MN1020; RRID:AB_223647
Anti-phospho-tau [AT180]	Thermo Fisher Scientific	MN1040; RRID:AB_223649
Anti-phospho-tau [AT270]	Thermo Fisher Scientific	MN1050; RRID:AB_223651
Anti-phospho-tau [PHF-1]	Provided by Peter Davies	RRID:AB_2315150
Anti-Actin	Sigma-Aldrich	A2066; RRID:AB_476693
Anti-Akt (pan) [40D4]	Cell Signaling	2920; RRID:AB_1147620
Anti-phospho AKT (Ser473) [D9E]	Cell Signaling	4060; RRID:AB_2315049
Anti-GSK3β (pan) [3D10]	Cell Signaling	9832; RRID:AB_10839406
Anti-phospho GSK3b (Ser9) [D85E12]	Cell Signaling	5558; RRID:AB_10013750
Anti-Mouse IgG – IRDye 800CW	Li-Cor Biosciences	925-32210; RRID:AB_2687825
Anti-Rabbit IgG – IRDye 800CW	Li-Cor Biosciences	925-32211; RRID:AB_2651127
Anti-human IgG – IRDye 800CW	Li-Cor Biosciences	926-32232; RRID:AB_10806644
Anti-Mouse IgG – IRDye 680CW	Li-Cor Biosciences	926-68070; RRID:AB_10956588
Anti-Rabbit IgG – IRDye 680CW	Li-Cor Biosciences	926-68071; RRID:AB_10956166
Anti-Mouse IgG-Alexa Fluor 488	Thermo Fisher Scientific	A28175; RRID:AB_2536161
Anti-Mouse IgG-Alexa Fluor 594	Thermo Fisher Scientific	A11032; RRID:AB_2534091
Anti-Rabbit IgG-Alexa Fluor 647	Thermo Fisher Scientific	A31573; RRID:AB_2536183
Anti-Mouse-Hrp	Thermo Fisher Scientific	31444; RRID:AB_228321
Anti-Human IgG-HRP	Southern Biotech	2087-05; RRID:AB_2795792

**Bacterial and virus strains**		

HSV-1 KOS	ATCC	VR-1493
BL21-(DE3)-Rosetta-pLysS	Novagen	70956
Stbl3	Invitrogen	C7373-03
DH5α	Thermo Fisher Scientific	18265017

**Biological samples**		

*Postmortem* brain samples	The Netherlands Brain Bank, Amsterdam, NL	www.brainbank.nl RRID:SCR_013841

**Chemicals, peptides, and recombinant proteins**		

Medium 199	Gibco	11310882
DMEM	Gibco	41965–039
DMEM/F12	Gibco	31330–038
Advanced DMEM/F12	Gibco	12634–010
HT-Supplement	Gibco	11067030
PFHM medium	Gibco	12040–077
FBS Xtra	Capricorn Scientific	FBS-16A
New Born Calf Serum (NBCS)	Gibco	16010167
mTeSR medium	Stem cell technol.	05850
Neural induction medium	Stem cell technol.	21103049
Neural basal medium	Thermo Fisher Scientific	21103049
Essential-8 medium	Thermo Fisher Scientific	A1517001
Geltrex	Thermo Fisher Scientific	A1413302
SB431542	Tocris	1614
Dorsomorphin	Tocris	3093/10
2-Mercaptoethanol	Thermo Fisher Scientific	21985023
Dispase	Thermo Fisher Scientific	17105041
Laminin	Sigma-Aldrich	L2020
Accutase	Thermo Fisher Scientific	00-4555-56
Optimem	Gibco	11058–021
Trypsin-EDTA Solution	Sigma-Aldrich	T3924
B-27 supplement w/o Vitamin A	Thermo Fisher Scientific	12587010
N2 supplement	Thermo Fisher Scientific	17502048
bFGF	Sigma-Aldrich	F0291
Fibronectin	Sigma-Aldrich	F2006
Poly-L-ornithine	Sigma-Aldrich	P3655
Doxycyclin-hydrochlorid	Sigma-Aldrich	D3447
GDNF	Sigma-Aldrich	G1777
Dibutyryl-cAMP	Santa Cruz Biotech.	Sc201567B
MEM NEAA	Gibco	11140–035
Sodium Pyruvate	Gibco	11360–039
Penicillin/Steptomycin	Sigma-Aldrich	P4333
L-Glutamin	Gibco	25030–024
Primocin	Thermo Fisher Scientific	NC9141851
Insulin	Sigma-Aldrich	I3536
Matrigel hESC-qualified matrix	Corning	354277
PBS	Gibco	14190–144
Human IgG	Sigma-Aldrich	I4506
KaryoMax Giemsa stain solution	Gibco	11529876
DMSO	Sigma-Aldrich	D8418
GeneJuice	Merck	70967
Lipofectamine 2000	Thermo Fisher Scientific	11668019
Lipofectamine 3000	Thermo Fisher Scientific	L3000008
Polybrene	Sigma-Aldrich	TR-1003-G
Hygromycin B	Thermo Fisher Scientific	10687010
Puromycin-Dihydrochlorid	Thermo Fisher Scientific	A1113802
NP40	Applichem	A1694
TX-100	Sigma-Aldrich	X100
Tween 20	Sigma-Aldrich	P1379
cOmplete EDTA-free protease inhibitor cocktail	Roche	11836170001
Phophatase Inhibitor cocktail 2	Sigma-Aldrich	P5726
DNase1	Roche	11284932001
NuPAGE 4–12% Bis-Tris gel (20 wells)	Invitrogen	WG1402BOX
NuPAGE 4–12% Bis-Tris gel (26 wells)	Invitrogen	WG1403BOX
NuPAGE LDS Sample buffer	Invitrogen	NP0007
NuPAGE MES Running Buffer	Invitrogen	NP0002
Nitrocellulose membrane (0.2 μm)	VWR	106000001
Ponceau S	Sigma-Aldrich	P3504
T4 PNK	Thermo Fisher Scientific	EK0031
FastDigest HindIII	Thermo Fisher Scientific	FD0504
FastDigest ClaI	Thermo Fisher Scientific	FD0144
FastDigest NdeI	Thermo Fisher Scientific	FD0583
FastDigest BamHI	Thermo Fisher Scientific	FD0054
BsmBI	NEB	R0580S
FastAP	Thermo Fisher Scientific	EF0651
Thiazolyl Blue Tetrazolium Bromide (MTT)	Sigma-Aldrich	M2128
Bovine Serum Albumin (BSA)	Sigma-Aldrich	A3294
Saponin	Sigma-Aldrich	84510
Fish gelatin	Sigma-Aldrich	G7041
Nonfat milk	Oxoid	LP0033B
ProLong Gold with DAPI	EMS	17984–24
Mowiol	Sigma-Aldrich	81381
Ribonucleotide triphosphate mix	NEB	N0466S
Creatine phosphate	Sigma-Aldrich	2380
Creatine kinase	Sigma-Aldrich	C3755
Instant Blue Coomassie Solution	Abcam	Ab119211
Carbencillin Disodium	Duchefa	C0109
Chloramphenicol	Sigma-Aldrich	C0378
Tryptone from casein	Serva	48647.02
Yeast extract	Sigma-Aldrich	92144
M9 medium	Studier et al.^83^	N/A
^13^C glucose	Cambridge Isotope Laboratories	CLM-420
^15^N-NH_4_Cl	Cambridge Isotope Laboratories	NLM-467
Isopropyl b-D-thiogalactopyranoside (IPTG)	Applichem	A008
CM-sepharose	GE-Healthcare	17-0719-10
Q-sepharose	GE-Healthcare	17-0510-10
Proclin300	Sigma-Aldrich	48912-U

**Critical commercial assays**		

OptEIA substrate	BD	555214
1-step Ultra TMB ELISA	Thermo Fisher Scientific	34029
DC Protein Assay Kit I	BioRad	5000111
IsoStrip	Roche	11493027001
PepSpots with 52 MIF peptides	JPT	N/A
DeadEnd Fluorometric TUNEL System	Promega	G3250
QuikChange Site-directed Mutagenesis Kit	Stratagene	200519

**Deposited data**		

Structure of MIF in complex with PAV-174	This paper	PDB:8CA0 (wwPDB deposition ID: D_1292128100) https://www.rcsb.org/structure/8CA0
Proteomics raw data of DRAC from resin 102 (PAV-645)	This paper	https://massive.ucsd.edu/ProteoSAFe/dataset.jsp?accession=MSV000099711 https://doi.org/10.25345/C56M33H2N

**Experimental models: cell lines**		

Vero Cell Line	ATCC	CCL-81; RRID:CVCL_0059
LUHMES Cell Line	ATCC	CRL-2927; RRID:CVCL_B056
TSM (exon10 + 16)V97	Sposito et al.^42^	RRID:CVCL_9S56
IMR90-1	WiCell	WISCi004-A; RRID:CVCL_C434
SH-SY5Y	DSMZ	ACC 209; RRID:CVCL_0019
SH-SY5Y-htau-P301S	This paper	N/A
SH-SY5Y-htau-P301S-MIF-knockdown	This paper	N/A
SH-SY5Y-htau-P301S-CUTA-knockdown	This paper	N/A
SH-SY5Y-htau-P301S-CRISPR-control	This paper	N/A
HEK293FT-MIF-ko	This paper	N/A
GP2-293	Clontech	RRID:CVCL_WI48
HEK-293FT	Clontech	RRID:CVCL_6911
P3U1	Sigma	85011417-1VL

**Experimental models: organisms/strains**		

Pigs	Struve Lab	1603, Enterprise St. Manning, IA 51455
Tau58/2 mice	Novartis	Van Eersel et al.^44^
MIF-ko mice	Provided by Jürgen Bernhagen, Munich	Fingerle-Rowson et al.^84^
PrP-ko mice - Prnp(ZH3/ZH3)	Provided by Adriano Aguzzi, Zurich	Nuvolone et al.^85^

**Recombinant DNA**		

pLHCX	TaKaRa	S1866
pLHCX-mod-MCS	This paper	N/A
pLNCX2	TaKaRa	631503
pLNCX2-MIF-wt-RESC	This paper	N/A
pLNCX2-MIF-C80W-RESC	This paper	N/A
pVSVG	Addgene	138479
lentiCRISPRv2	Addgene	52961
pMDLG/pRRE	Addgene	12251
pRSV_Rev	Addgene	12253
pMD2.G	Addgene	12259
pCMV6-entry MIF	Origene	NM_002415
pET11a	Novagen	69436
pET11a-hMIF	This paper	N/A
pET11a-hMIF-C80W	This paper	N/A

**Software and algorithms**		

Fiji software (ImageJ 2.0.0)	NIH, USA	RRID:SCR_002285
Prism (version 6 & 9.4)	GraphPad Software	RRID:SCR_005375
sgRNA Design tool (Zhang Lab)	MIT-CRISPR server	http://crispr.mit.edu
Saffold-2 Viewer	Proteome Software	https://www.proteomesoftware.com
NMR^2^ software	ETH Zürich	N/A
Cyana	L.A. systems Inc.	https://www.las.jp/english/products/cyana.html
Relax		http://www.nmr-relax.com
Chimera		https://www.cgl.ucsf.edu/chimera/
Image Studio (version 2.1)	LI-COR Biosciences	RRID:SCR_015795

**Other**		

Misonix-S-4000 Ultrasonic water bath	Misonix	N/A
Tecan Safire	Tecan	N/A
Apotome.2 microscope	Zeiss	N/A
Leica SP8 confocal system	Leica	N/A
700 MHz Avance *Neo* spectrometer	Bruker	N/A
600 MHz Avance III spectrometer	Bruker	N/A
1200 series HPLC system	Agilent	N/A
TSKgel G2000SWXL	Tosoh Biosciences	N/A
miniDawn Treos spectrometer	Wyatt	N/A
Tank Blot system	BioRad	1703939
LI-COR Odyssey CLX	LI-COR Biosciences	N/A
96-well-Immuno Maxisorp plates	Nunc	44-2404-21
Amicon Ultra-4 10 kDa cut-off	Merck	UFC801024
0.45 μm filter	VWR	514–0063

**Oligonucleotides**		

Modified MCS _fw: 5′aaaaaaAAGCTTACC GGTCTCGAGGCGGCCGCGGCCAAAAAGG CCGGATCCGTTAACACCAAAAAATGGCAC GTGGCCGGCACGCGTGGGCCCGTCGACA TCGATaaaaaa-3′	Biomers	N/A
Modified MCS_rev:5′-ttttttatcgatGTCGACGGG CCCACGCGTGCCGGCCACGTGCCATTTTTT GGTGTTAACGGATCCGGCCTTTTTGGCCGC GGCGCCTCGAGACCGGTaagctttttttt-3′	Biomers	N/A
Tau5 P301S FOR: 5′ AAACACGTCTCG GGAGGCGGC-3′	Biomers	N/A
Tau6 P301S REV: 5′- GCCGCCTCCCGAG ACGTGTTT-3′	Biomers	N/A
MIF-ko-fw: 5′:CACCGAGCTCGGAGAG GAACCCGTC-3′	Biomers	N/A
MIF-ko-rev: 5′:AAACGACGGGTTCCT CTCCGAGCTC-3′	Biomers	N/A
CUTA-ko-fw:5′:CACCGCCTTGGCGACC TTCTCGTTG -3′	Biomers	N/A
CUTA-ko-rev: 5′: AAACCAACGAGAAGGT CGCCAAGGC -3′	Biomers	N/A
CRISPRctrl_fw: 5′CACCGGCACTCACATCGCTACATCA-3′	Biomers	N/A
CRISPRctrl_rev: 5′ AAACTGATGTAGCGATGTGAGTGCC-3′	Biomers	N/A
pET11a MIF-fw: 5′-AAAAAACATATG CCGATGTTCATCGTAAAC -3′	Biomers	N/A
pET11a MIF-rev: 5′-AAAAAAGGATCCTTA GGCGAAGGTGGAG-3′	Biomers	N/A
MIF-C80W-fw: 5′-CCTACAGCAAGCTGCT GTGGGGCCTGCTGGCCGAGCGCC-3′	Biomers	N/A
MIF-C80W-rev:5′-GGCGCTCGGCCAGCA GGCCCCACAGCAGCTTGCTGTAGG-3′	Biomers	N/A
MIF_HindIII_fw: 5′- AAAAAA AAGCTT ATGCCGATGTTCATCGTAAAC	Biomers	N/A
MIF_ClaI_rev: 5′- AAAAAA ATCGAT TTAGGCGAAGGTGGAGTTG	Biomers	N/A
MIF-ko-rescue-fw: 5′-CGCGCCTCCGTGCCGG ATGGATTTTTGTCCGAGCTCACCCAGCAGC-3′	Biomers	N/A
MIF-ko-rescue-rev: 5′- GCTGCTGGGTGAGCTCG GACAAAAATCCATCCGGCACGGAGGCGCG -3′	Biomers	N/A

### Experimental model and study participant details

#### Animals and human samples and ethics

TgTau58/2 mice^44^ that express the human 0N4R tau isoform with the P301Smutation under control of the mouse Thy1.2 promoter were a kind gift from Novartis (Boston, USA). Experimental animals were two months old, male, and heterozygous for the transgene. Wildtype littermates on C57BL/6J background served as control. The mice were maintained in pathogen-free conditions in the Animal Facility at the University of Düsseldorf, Germany, fed *ad libitum* with standard laboratory chow and water in ventilated cages under a 12h light/dark cycle. All experiments were in conformity with the Animal Protection Law approved by local authorities (LANUV NRW, Recklinghausen, Germany).

Studies investigating the pharmacokinetics of PAV-617 were performed by Vipragen Biosciences Inc in female Balb/C mice. The animal care committee approval numbers for the studies are: M-Tox: VIP/IAEC/41/2016 and PK: VIP/IAEC/316/2021.

Human brain samples were obtained from the The Netherlands Brain Bank, Netherlands Institute for Neuroscience, Amsterdam (www.brainbank.nl). The use of these samples for this study was approved by the Ethics Commission of the Medical Faculty of the Heinrich Heine University, Düsseldorf. Human brain samples for AD and matched healthy controls (HC) were balanced for age and gender; AD: female (f) 72 years old (y), f 68 years, f, 84 years, f, 86 years, male (m) 87 years, f 90 years, m 71years, m 70 years, f 89 years, m 81 years. HC: f 60 years, f 75 years, f 72 years, f 78 years, m 90 years, m 73 years, f 88 years, f 91 years, m 83 years, m 102 years. Please see Figure S6A for information on gender and age. Group assignment was performed according to Braak and Thal staging (see Figures S6A and S6B).

#### Cell culture

Vero (ATCC), GP2-293 (Clontech) and HEK-293FT cells (Clontech) were cultured in DMEM (Gibco) supplemented with 10% FBS xtra (Capricorn Scientific), 1 mM sodium pyruvate (Gibco), and 1% (v/v) penicillin/streptomycin (Sigma). Human neuroblastoma SH-SY5Y cells were obtained from the DSMZ (Leibniz Institute DSMZ-German Collection of Microorganisms and Cell Cultures, Braunschweig, Germany) and cultured in DMEM/F12 Medium (Gibco) supplemented with 10% FBS xtra (Capricorn Scientific), 1x non-essential amino acids (MEM NEAA, Gibco), 1% (v/v) penicillin/streptomycin (Sigma). LUHMES cells (ATCC) were maintained in a proliferative state in advanced DMEM/F12 (Gibco) supplemented with 1% N2 (Thermo Fisher Scientific), 1% (v/v) penicillin/streptomycin (Sigma), 2 mM L-glutamine (Gibco) and 40 ng/mL bFGF (basic recombinant human fibroblast growth factor ; Sigma) in flasks coated first with 50 μg/mL poly-L-ornithine (Sigma) and then with 1 μg/mL fibronectin (Sigma). For differentiation into post-mitotic neurons the cells were cultured in advanced DMEM/F12 supplemented with 1 μg/mL doxycyclin (Sigma), 2 ng/mL GDNF (glial cell line-derived neurotropic factor; Sigma), and 1 mM dibutyryl-cAMP (Santa Cruz Biotechnology) in coated flasks for 2 days according to Scholz et al.^86^ The cells were then washed with PBS and trypsinized with Trypsin-EDTA solution (Gibco), counted and seeded on target plates in differentiation medium.

Culture and differentiation of TSM(exon10 + 16)V97 iPSC were performed as previously described, and all reagents were purchased from Thermo Fisher Scientific unless otherwise stated.^87^ iPSC were cultured on geltrex matrix (Thermo Fisher Scientific) coated plates in Essential-8 media. iPSC were grown to 100% confluency prior to neuronal induction using dual SMAD inhibition as described previously.^88^ Briefly, cells were cultured for 10 days in neural induction media (N2B27 containing 10 μM SB431542 (Tocris) and 1 μM dorsomorphin (Tocris)). N2B27 media consists of a 1:1 mixture of Dulbecco’s modified Eagle's medium F12 (DMEM-F12) and Neurobasal, supplemented with 0.5x *N*-2, 1x B-27, 2.5 μg/mL insulin (Sigma), 1 mM L-glutamine, 0.5x MEM NEAA solution, 50 μM 2-mercaptoethanol, 25 U ml−1 Penicillin-Streptomycin. At days 10 and 18, neuronal rosettes were passaged using dispase and plated in laminin-coated wells in N2B27 media. Media was changed every other day. The final passage was performed at day 35 using accutase, and cells were plated at a final density of 50,000 cells per cm^2^ on laminin and poly-L-ornithine coated wells and maintained in N2B27 media until treatment. Mature neurons were treated at 70–90 DIV and harvested 24-48h post-treatment.

Human brain organoids were generated from a commercially available hiPSC line (IMR90-1, Wi Cell). The hiPSCs were maintained on mTeSR medium, under non differentiating conditions for initial expansion. To differentiate into the neural lineage for organoid generation, the hiPSCs were allowed to self-aggregate in 96-well plates for five days using Neural induction medium (Stem cell technologies) as described earlier.^89^ The aggregated neurospheres were further matured to form brain organoids in spinner flaks, in a medium composed of DMEM-F12: Neural Basal medium (1:1), 1:100 B27 v/o Vitamin A (Thermo Scientific), 1:200 N2 (Thermo Scientific), 1:100 L-Glutamine (Gibco), 100 μg/mL Primocin (Thermo Fisher Scientific), 0.05% Insulin (Sigma Aldrich) and 0.1% Matrigel (Corning). All cells were tested at regular time intervals for mycoplasma contamination and cultured at 37°C with 5% CO_2_.

### Method details

#### Chemical synthesis

##### 1-Ethyl-9-methylphenothiazin-5-ium tetraiodide hydrate

a. N-Acetyl-2-ethylaniline (2): commercial 2-ethylaniline (1) (50mL, 0.40 mol) was dissolved in acetic anhydride (160mL, 1.70 mol) and stirred at room temperature for 2h. Then the reaction mixture was poured into H_2_O, the whole was extracted with ethyl acetate (2x200mL). The combined organic extracts were washed with 5% aqueous NaHCO_3_, brine, dried (K_2_CO_3_), filtered and concentrated to provide the title compound as a white solid (60.0 g, 92%).
b. N-Acetyl-2-ethyl-2′-methyldiphenylamine (4): a mixture of the N-acetyl-2-ethylaniline (35.0 g, 215 mmol), anhydrous Cs_2_CO_3_ (70.0 g, 215 mmol), CuBr (2.86 g, 20 mmol), KI (3.33 g, 20 mmol) and 2-bromotoluene (3) (78mL, 640 mmol) was stirred and heated at 175-180^0^C under an argon atmosphere for 48h. After cooling the reaction mixture was poured into ice-H_2_O and extracted with ethyl acetate (2x200mL), the combined organic extracts were washed with brine, dried over anhydrous K_2_CO_3_, filtered and concentrated to dryness. The obtained crude material was purified by flash chromatography (using ethyl acetate - hexane as an eluent) to afford the N-acetyl-2-ethyl-2′-methyldiphenylamine (35.4 g, 65%).
c. 2-Ethyl-2′-methyldiphenylamine (5): a solution of the N-acetyl-2-ethyl-2′-methyldiphenyl-amine (32.5 g, 128 mmol) in 10% KOH (72 g, 1.28 mol)/EtOH (120mL) was stirred and refluxed for 6 h, then poured into H_2_O. The mixture was extracted with ethyl acetate (2x100mL). The combined organic layers were washed with brine, dried (Na_2_SO_4_), filtered and concentrated to dryness, gave dark red oil (21.1g, 78%).
d. 1-Ethyl-9-methyl-10H-phenothiazine (6): to a 2-ethyl-2′-methyldiphenylamine (3.0 g, 14.2 mmol), sulfur (909 mg, 28.4 mmol) and iodine (601 mg, 4.7 mmol) were added. Vial was charged with balloon for discharge. The heating block was preheated (150^0^C). The vial was heated on the heating block and after 15 min temperature was increased to 210^0^C, reaction mixture was stirred and heated for an additional 1 h. The mixture was allowed to cool to 90^0^C. The dark solid material was dissolved in mixture methanol/chloroform and purified by flash chromatography (ethyl acetate - hexane as an eluent) to afford the desired product (790 mg, 23%).
e. 1-Ethyl-9-methylphenothiazin-5-ium tetraiodide hydrate (7): a solution of 1-ethyl-9-methyl-10H-phenothiazine (4.83g, 20 mmol) in anhydrous chloroform (50mL) was stirred at 5^0^C and the solution of iodine (15.25 g, 60 mmol) in CHCl_3_ (300mL) was added drop wise over 3h. The resulting dark solution was stirred for an additional 3h at 5^0^C, monitored by TLC. After the disappearance of the starting material, the resulting precipitate was filtered, washed with a copious amount of chloroform, dried overnight in vacuum to afford a dark solid (9.18 g, 60%).

##### 3,7-Di- (4-methylpiperazin-1-yl)-1-ethyl-9-methylphenothiazin-5-ium iodide (PAV-152)



A solution of 1-ethyl-9-methylphenothiazin-5-ium tetraiodide hydrate (50 mg, 0.07 mmol) in methanol (10mL) and 1-methylpiperazine (30 mg, 0.3 mmol) was stirred for 2 h at room temperature. The resulting mixture was concentrated to dryness and purified by flash chromatography using the methanol-chloroform gradient to provide the title compound.

##### 3,7-Di-(morpholin-1-yl)-1-ethyl-9-methylphenothiazin-5-ium iodide (PAV-215)



A solution of 1-ethyl-9-methylphenothiazin-5-ium tetraiodide hydrate (50 mg, 0.07 mmol) in methanol (10mL) and morpholine (0.05 mL, 0.5 mmol) was stirred for 1 h at room temperature. The resulting mixture was concentrated to dryness and purified by flash chromatography using the methanol-chloroform gradient to provide the title compound.

##### 3,7-Di-(4-(dimethylamino)piperidin-1-yl)-1-ethyl-9-methylphenothiazin-5-ium iodide (PAV-251)



A solution of 1-ethyl-9-methylphenothiazin-5-ium tetraiodide hydrate (382 mg, 0.5 mmol) in acetonitrile (10mL) and 4-(dimethylamino)piperidine (192 mg, 1.5 mmol) was stirred for 1 h at room temperature. The resulting mixture was concentrated to dryness and purified by flash chromatography using the methanol-chloroform gradient to provide the title compound.

##### 3,7-Di-(tiomorpholin-1-yl)-1-ethyl-9-methylphenothiazin-5-ium iodide (PAV-269)



A solution of 1-ethyl-9-methylphenothiazin-5-ium tetraiodide hydrate (191 mg, 0.25 mmol) in mixture methanol and acetonitrile (1:1) (10mL) and tiomorpholine (0.1 mL, 1.0 mmol) was stirred for 1 h at room temperature. The resulting mixture was concentrated to dryness and purified by flash chromatography using the methanol-chloroform gradient to provide the title compound.

##### 3,7-Di(piperazin-1-yl)-1-ethyl-9-methylphenothiazin-5-ium trifluoroacetate (PAV-382)



A solution of 1-ethyl-9-methylphenothiazin-5-ium tetraiodide hydrate (153 mg, 0.2 mmol) in mixture methanol and acetonitrile (1:1) (10mL) and N-Bocpiperazine (186 mg, 1.0 mmol) was stirred for 1 h at room temperature. The resulting mixture was concentrated to dryness and dissolved in DCM. TFA (1 mL) was added with stirring. After 30 min mixture was concentrated and purified by prep-HPLC to provide the title compound.

##### 3,7-Di(homopiperazin-1-yl)-1-ethyl-9-methylphenothiazin-5-ium trifluoroacetate (PAV-352)



A solution of 1-ethyl-9-methylphenothiazin-5-ium tetraiodide hydrate (153 mg, 0.2 mmol) in mixture methanol and acetonitrile (1:1) (10mL) and N-Bochomopiperazine (200 mg, 1.0 mmol) was stirred for 1 h at room temperature. The resulting mixture was concentrated to dryness and dissolved in DCM. TFA (1 mL) was added with stirring. After 30 min mixture was concentrated and purified by prep-HPLC to provide the title compound.

##### 3,7-Di(pyrrolidine-1-yl) 1-ethyl-9-methyl-phenothiazin-5-ium iodide (PAV-174)



To the stirred mixture of 1-ethyl-9-methylphenothiazin-5-ium tetraiodide hydrate (383 mg, 0.5 mmol) in methanol (20mL) pyrrolidine (142 mg, 2.0 mmol) was added dropwise. The resulting mixture was stirred at room temperature 1 h, concentrated to dryness. Compound was purified with prep-HPLC.

##### 3-(Dimethylamino)-1-ethyl-9-methyl-7-(4-(trifluoromethylsulfonyl)piperazin-1-yl)phenothiazin-5-ium iodide (PAV-173)



To the stirred mixture of 1-ethyl-9-methylphenothiazin-5-ium tetraiodide hydrate (383 mg, 0.5 mmol) (52) in anhydrous CHCl_3_ (20mL) dimethylamine (0.5mL, 1.0 mmol, 2M solution in THF) was added dropwise over 0.5 h. The resulting mixture was stirred at room temperature 1 h and concentrated to dryness.

A solution of 3-(dimethylamino)-1-ethyl-9-methylphenothiazin-5-ium triiodide (100 mg, 0.15 mmol) in methanol (10mL), (piperazin-1-yl)trifluoromethyl sulfone hydrochloride (115 mg, 0.45 mmol) and triethylamine (0.5mL) was stirred for 2 h at room temperature. The resulting mixture was concentrated to dryness and purified by flash chromatography using the methanol-chloroform gradient to provide the title compound.

##### 1-Ethyl-9-methyl-7-(piperidin-1-yl)-3-(4-(Bocamino)piperidin-1-yl)phenothiazin-5-ium trifluoroacetate (PAV-322)



To the stirred mixture of 1-ethyl-9-methylphenothiazin-5-ium tetraiodide hydrate (153 mg, 0.2 mmol) in anhydrous CHCl3 (10mL) 4-Bocaminopiperidine (60 mg, 0.3 mmol) was added with stirring. The resulting mixture was stirred at room temperature overnight, concentrated to dryness. A solution of 1-ethyl-9-methyl-3-(4-Bocamino)piperidin-1yl)phenothiazin-5-ium triiodide (100 mg, 0.12 mmol) in mixture acetonitrile-methanol (1:1) (10mL) and piperidine (0.03 mL, 0.3 mmol) was stirred for 1 h at room temperature. The resulting mixture was concentrated to dryness and purified by flash chromatography using the methanol-chloroform gradient. Product was concentrated to dryness and dissolved in DCM. TFA (1 mL) was added with stirring. After 30 min mixture was concentrated and purified by prep-HPLC to provide the title compound.

##### 1-Ethyl-9-methyl-7-morpholino-3-(4-Bocaminopiperidin-1-yl)phenothiazin-5-ium iodide (PAV-395)



To the stirred mixture of 1-ethyl-9-methylphenothiazin-5-ium tetraiodide hydrate (153 mg, 0.2 mmol) in anhydrous CHCl_3_ (10mL) 4-Bocaminopiperidine (60 mg, 0.3 mmol) was added with stirring. The resulting mixture was stirred at room temperature overnight, concentrated to dryness. A solution of 1-ethyl-9-methyl-3-(4-Bocaminopiperidin-1yl)phenothiazin-5-ium triiodide (165 mg, 0.2 mmol) in methanol (10mL) and morpholine (17.4 mg, 0.2 mmol) was stirred for 4 h at room temperature. The resulting mixture was concentrated to dryness and purified by flash chromatography using the methanol-chloroform gradient.

##### 1-Ethyl-9-methyl-7-tiomorpholino-3-(4-Boc-1,4-diazepane-1-yl)phenothiazin-5-ium iodide (PAV-396)



To the stirred mixture of 1-ethyl-9-methylphenothiazin-5-ium tetraiodide hydrate (780 mg, 1.0 mmol) in anhydrous CHCl_3_ (15 mL) 1-Boc-1,4-diazepane (400 mg, 2.0 mmol) was added at room temperature. The resulting mixture was stirred at this temperature for 4 h. Solvent was removed under vacuum. A solution of 1-ethyl-9-methyl-3-(4-Boc-1,4-diazepane-1-yl)phenothiazin-5-ium triiodide (165 mg, 0.2 mmol) in acetonitrile (10mL) and tiomorpholine (72 mg, 0.8 mmol) was stirred for 4 h at room temperature. The resulting mixture was concentrated to dryness and purified by flash chromatography using the methanol-chloroform gradient. Structure of compounds PAV-173, PAV-322, PAV-395 and PAV-396 was confirmed by stereospecific synthesis by the method described below for PAV-645.

##### 1,9-Diethylphenothiazin-5-ium tetraiodide hydrate

The same scheme and procedures like for 1-ethyl-9-methylphenothiazinium salt. Compound 3 is 2-ethylbromobenzene.



##### 1,9-Diethyl-3-(1,4-diazepane-1-yl)-7-dimethylaminophenothiazin-5-ium iodide (PAV-493)



To the stirred mixture of 1,9-diethylphenothiazin-5-ium tetraiodide hydrate (3.9 g, 5.0 mmol) in anhydrous CHCl3 (100 mL) 1-Boc-1,4-diazepane (1.2 g, 6.0 mmol) was added at room temperature. The resulting mixture was stirred at this temperature for 2 h. Solvent was removed under vacuum. A solution of 1,9-diethyl-3-(4-Boc-1,4-diazepane-1-yl)phenothiazin-5-ium triiodide in mixture methanol-acetonitrile (1:1) (150mL) and dimethylamine (10 mL 2 M sol. in THF) was stirred for 1 h at room temperature. The resulting mixture was concentrated to dryness and purified by flash chromatography using the methanol-chloroform gradient. Product (300 mg) was concentrated to dryness. HCl (5 mL 4 M solution in 1,4-dioxane) was added with stirring. After 30 min mixture was concentrated and purified by prep-HPLC to provide the title compound.

##### 1-Ethyl-7-(piperazin-1-yl)-3-diethylaminophenothiazin-5-ium chloride (PAV-645)



2-Amino-5-bromo-3-ethyl-benzenethiol: 6-Bromo-4-ethyl-1,3-benzothiazol-2-amine (2.0 g, 7.7 mmol) was added to a solution of KOH (13.5 g, 240 mmol) and H2O (25 mL) and the reaction mixture was heated to 150°C overnight. The reaction was monitored by LCMS for consumption of starting material. At the completion of the reaction, the reaction was allowed to cool to RT and ice bath was added as the mixture was slowly neutralized with conc. HCl to pH = 6. The solid was filtered off and dried under vacuum overnight. Both the solid and the filtrate were washed with Et2O and the organic layers were combined. The resulting organic layer was washed with brine, dried over MgSO4, filtered and evaporated to give the product, which was used without further purification. LCMS, M + H = 232.0.

4-Bromo-2-(4-chloro-2-nitro-phenyl)sulfanyl-6-ethyl-aniline: 2-Amino-5-bromo-3-ethylbenzenethiol (2.3 g, 10 mmol) was combined with 1,4-dichloro-2-nitrobenzene (2.03 g, 10.6 mmol), Cs2CO3 (10.29 g, 31.6 mmol) and acetonitrile (50mL) and stirred at room temperature overnight. The starting material was monitored by TLC. At the completion of the reaction, the reaction was filtered, and the solvent evaporated to give a residue. The residue was purified by flash silica gel chromatography to give the desired product. LCMS: M + H = 388.

N-[4-Bromo-2-(4-chloro-2-nitro-phenyl)sulfanyl-6-ethyl-phenyl]formamide: 4-Bromo-2-ethyl-6- (4-chloro-2-nitro-phenyl)sulfanyl-aniline (14.6 g, 39.22 mmol) was dissolved in formic acid (50mL) and heated at 100°C overnight. The reaction was allowed to cool to RT and ice water was added, keeping the temperature near 0°C. The resulting solid was filtered off, washed with cold water and dried overnight under vacuum. LCMS, M + H = 416.

3-Bromo-7-chloro-1-ethyl-10H-phenothiazine: N-[4-Bromo-2-ethyl-6-(4-chloro-2-nitrophenyl) sulfanyl-phenyl]formamide was combined with acetone (20 mL) and heated to reflux and an alcoholic solution of KOH (0.7 g,12.4 mmol) in EtOH (20mL) was added. The resulting solution was refluxed for 30 min to 1 h. Another portion of alcoholic KOH (0.7 g,12.4 mmol) was added and the resulting reaction was refluxed for 4 h. The mixture was allowed to cool to room temperature. The solvent was evaporated, and the residue was extracted with CHCl3 and brine. The combined organic layers were dried over MgSO4, filtered, and evaporated to give a residue. The crude material was purified by flash silica gel chromatography to give the desired compound. LCMS, M + H = 341 *tert*-Butyl 3-bromo-7-chloro-1-ethyl-phenothiazine-10-carboxylate: 3-Bromo-1-ethyl-7-chloro-10H-phenothiazine (1.5 g, 4.40 mmol), was combined with Boc2O (1.92 g, 8.81 mmol), DMAP (0.54 g, 4.40 mmol), and acetonitrile (30 mL). The resulting reaction mixture was stirred and, heated to reflux overnight. After cooling the reaction mixture was concentrated to dryness and purified on the ISCO using EtOAc/Hexanes gradient to afford the desired compound as a waxy solid. M + H = 441*tert*-Butyl 7-chloro-3-diethylamino-1-ethylphenothiazine-10-carboxylate: To a mixture of Na t-OBu (23 mg, 0.237mmol), Pd(dba)2 (2.3 mg, 0.004mmol), BINAP (2.5 mg, 0.004mmol), diethylamine (17 mg, 0.237mmol) and the *tert*-butyl 3-bromo-7-chloro-phenothiazine-10-carboxylate (85 mg, 0.206mmol) was added dioxane (2.5mL) (all in a flame dried screw top vial). The mixture was stirred, under argon, at 100°C for 4h (take an aliquot after 2h and check for completeness). Upon completion the reaction was cooled diluted with dioxane (5mL) and filtered through a small Celite plug. The filtrate was rotary evaporated to dryness and the residue purified by the flash-chromatography. Yield 75mg (91%) LCMS, M + H = 433.

*tert*-butyl 7-(N-Bocpiperazin-1-yl)-3-diethylamino-1-ethylphenothiazine-10-carboxylate: To a mixture of Na t-OBu (23 mg, 0.237mmol), PdRuPhos (3 mg, 0.004 mmol), RuPhos (2 mg, 0.004 mmol), N-Bocpiperazine (47 mg, 0.237mmol) and *tert*-Butyl 7-chloro-3-diethylamino-1-ethylphenothiazine-10-carboxylate (83mg, 0.2mmol) was added THF (2.5mL) (all in a flame dried screw top vial). The mixture was stirred under argon at 85°C for 4h (take an aliquot after 2h and check for completeness). Upon completion the reaction was cooled diluted with 10mL of THF and filtered through a small Celite plug. The filtrate was rotary evaporated to dryness and the residue purified on the ISCO. Yield 72mg (80%) LCMS, M + H = 583.

3-Diethylamino-7-(piperazin-1-yl)-1-ethylphenothiazin-5-ium: A 10mg sample of *tert*-butyl 3-diethylamino-7-(piperazin-1-yl)-1-ethylphenothiazine-10-carboxylate was treated with 200ul of 4N HCl in dioxane for 1h with stirring. The mixture was then rotary evaporated to dryness. Yield = 7.5 mg as HCl salt. LCMS, M + H = 418.



A solution of 3-Diethylamino-7-(piperazin-1-yl)-1-ethylphenothiazin-5-ium chloride (13,6 mg, 0.03 mmol), DIEA (60 μL, 034 mmol) and DMAP (2 mg) in 2 mL of DMF was added to 3 mL of AffiGel-10 resin (Bio-Rad) in an Econo-Pac Chromatography Column. After agitating overnight at room temperature, the resin was washed 3x with 4 mL of DMF and then stored under 3 mL of i-propanol.

PAV-802 is methylene blue, a commercially available compounds which was purchased from Aldrich fine chemicals, Inc.

##### Synthesis of 3,7-Di(pyrrolidine-1-yl)phenothiazine-5-ium iodide (PAV-617)

Phenothiazin-5-ium tetraiodide hydrate.



Phenothiazin-5-ium tetraiodide hydrate: a solution of phenothiazine (4.98 g, 25 mmol) in anhydrous chloroform (50 mL) was stirred at 5^0^C and the solution of iodine (12.7 g, 50 mmol) in CHCl_3_ (250 mL) was added dropwise over 4h. The resulting dark solution was stirred for an additional 3h at 5^0^C, monitored by TLC. After the disappearance of the starting material, the resulting precipitate was filtered, washed with a copious amount of chloroform, dried overnight in vacuo to afford a dark solid (13.9 g, 74%).

3,7-Di(pyrrolidine-1-yl)phenothiazine-5-ium iodide.



A solution of phenothiazin-5-ium tetraiodide hydrate (2.8 g, 3.6 mmol) in mixture acetonitrile/methanol (50 mL) and pyrrolidine (710 mg, 10 mmol) was stirred for 4 h at room temperature. The resulting mixture was concentrated to dryness and purified by flash chromatography using the methanol-chloroform gradient to provide the title compound.

##### Synthesis of resin 428 [resin for 3-(3-aminopyrrolidin-1-yl)-7-(pyrrolidin-1-yl) phenothiazine-5-ium iodide]

a. Phenothiazin-5-ium tetraiodide hydrate: A solution of phenothiazine (4.98 g, 25 mmol) in anhydrous chloroform (50 mL) was stirred at 5 C and the solution of iodine (12.7 g, 50 mmol) in CHCl3 (250 mL) was added dropwise over 4h. The resulting dark solution was stirred for an additional 3h at 5 C, monitored by TLC. After the disappearance of the starting material, the resulting precipitate was filtered, washed with a copious amount of chloroform, dried overnight in vacuo to generate a dark solid (13.9 g, 74%).
b. 3-(3-Bocaminopyrrolidin-1-yl) phenothiazin-5-ium iodide: a solution of phenothiazin-5-ium tetraiodide hydrate (2.8 g, 3.6 mmol) in chloroform (20 mL) and 3-Bocaminopyrrolidine (745 mg, 4.0 mmol) was stirred for 1 h at room temperature. The resulting mixture was concentrated to dryness, rinse 3 times with hexane (3x10 mL) to extract excess of nonreacted amine and used without purification.
c. 3-(3-Bocaminopyrrolidin-1-yl)-7-(pyrrolidin-1-yl) phenothiazin-5-ium iodide: to the stirred solution of 3-(3-Bocaminopyrrolidin-1-yl) phenothiazin-5-ium iodide (510 mg, 1.0 mmol) in methanol (10mL) pyrrolidine (85 mg, 0.1 mL, 1.2 mmol) was added all at once with vigorous stirring at room temperature. The resulting mixture was stirred at room temperature 1h, concentrated to dryness. The crude product was purified by reverse flash chromatography (water-acetonitrile as an eluent) to provide the title compound.
d. 3-(3-Aminopyrrolidin-1-yl)-7-(pyrrolidin-1-yl) phenothiazin-5-ium iodide hydrochloride: to the stirred solution of 3-(3-Bocaminopyrrolidin-1-yl)-7-(pyrrolidin-1-yl) phenothiazin-5-ium iodide (579 mg, 1 mmol) in methanol (5mL) HCl (3 M solution in methanol, 5 mL) was added all at once with vigorous stirring at room temperature. The resulting mixture was stirred at room temperature 1h, concentrated to dryness. The crude product was purified by reverse flash chromatography (water-acetonitrile as an eluent) to provide the title compound.
e. To a solution of Affi-Gel (Bio-Rad, 10 mL) in a solid phase synthesis tube with frit was added a solution of 3-(3-aminopyrrolidin-1-yl)-7-(pyrrolidin-1-yl) phenothiazin-5-ium iodide hydrochloride (57 mg, 0.11 mmol) and DIEA (1.0 mL) in isopropyl alcohol (4 mL) and the tube was put in a shaker for 12 h. Excess reagents were drained and the resin was washed with isopropyl alcohol (3×) and then saved in isopropyl alcohol.

The loading density is 15umol per 1mL of resin, so coupling is done in slight resin site excess. Remaining attachment sites are blocked with ethanolamine and the final resin washed extensively with isopropyl alcohol for storage and with 100 bed volumes of column buffer before use.

##### Synthesis of resin 435 [resin for 2-aminomethyl-3,7-di(pyrrolidin-1-yl) phenothiazin-5-ium iodide]

a. 2-Aminomethylphenothiazine: To a solution of 2-cyanophenothiazine (2.24 g, 10 mmol) in anhydrous THF (50mL) was added lithium aluminum hydride (LAH) (2M solution in THF, 40 mmol, 20 mL) at room temperature. The reaction mixture was stirred for 2h (monitoring for completion by TLC and LCMS). The volume of solution was reduced in half by rotary evaporation followed by an equivalent volume of diethyl ether. The reaction was then cooled to 0°C and with rapid stirring was treated dropwise with water (1.5mL) over 5 min. Stirring was continued for 10 min followed by the slow addition of 15% aqueous sodium hydroxide (1.5mL). Stirring was continued for 10 min after which water (5 mL) was added and the mixture was warmed to room temperature. Anhydrous MgSO4 was added and stirring continued for 15 min followed by filtration to remove salts. The solvent was removed and product was purified by flash chromatography (85% yield).
b. 2-Bocaminomethylphenothiazine: 2-Aminomethylphenothiazine (4.0 g, 17.5 mmol) was dissolved in dichloromethane (DCM) (100 mL) and a solution of Boc anhydride (3.85 g, 17.5 mmol), also in DCM (20 mL), was added with vigorous stirring. Reaction progress was monitored by TLC. The solvent was removed under vacuum to afford product. Compound was purified by flash chromatography (95% yield).
c. 2-Bocaminomethylphenothiazin-5-ium tetraiodide hydrate: 2-Bocaminomethyl-phenothiazine (328 mg, 1 mmol) was dissolved in chloroform (5 mL) and a solution of iodine (1.27 g, 5 mmol) also in chloroform (30 mL) was added to it drop wise at r.t. for 4 h with vigorous stirring. Reaction progress was monitored by TLC. A black colored precipitate was filtered, washed with copious amount of chloroform and dried under vacuum to afford product.
d. 2-Bocaminomethyl-3,7-di(pyrrolidin-1-yl) phenothiazin-5-ium iodide: To a stirred solution of 2-Bocaminomethylphenothiazin-5-ium tetraiodide hydrate (426 mg, 0.5 mmol) in chloroform (10mL) pyrrolidine (85 mg, 0.1 mL, 1.2 mmol) was added all at once with vigorous stirring at room temperature. The resulting mixture was stirred at room temperature 3h and concentrated to dryness. The crude product was purified by reverse flash chromatography (water-acetonitrile as an eluent) to provide the title compound.
e. 2-Aminomethyl-3,7-di(pyrrolidin-1-yl) phenothiazin-5-ium iodide hydrochloride: To a stirred solution of 2-Bocaminomethyl-3,7-di(pyrrolidin-1-yl) phenothiazin-5-ium iodide (465 mg, 1 mmol) in methanol (5mL) was added in one portion HCl (3 M solution in methanol, 5 mL) with vigorous stirring at room temperature. The resulting mixture was stirred at room temperature 1h and concentrated to dryness. The crude product was purified by reverse flash chromatography (water-acetonitrile as an eluent) to provide the title compound.
f. To a solution of Affi-Gel (Bio-Rad, 10 mL) in a solid phase synthesis tube with frit was added a solution of 2-aminomethyl-3,7-di(pyrrolidin-1-yl) phenothiazin-5-ium iodide hydrochloride (54 mg, 0.11 mmol) and DIEA (1.0 mL) in isopropyl alcohol (4 mL) and the tube was put on a shaker for 12 h. Excess reagents were drained and the resin was washed with isopropyl alcohol (3×) and then stored in isopropyl alcohol.

The loading density is 15umol per 1mL of resin, so coupling is done in slight resin site excess. Remaining attachment sites are blocked with ethanolamine and the final resin washed extensively with isopropyl alcohol for storage and with 100 bed volumes of column buffer before use.

#### Generation of monoclonal antibodies

Monoclonal antibody (mAB) 17F3 was generated by immunizing a PrP knockout mouse^85^ with recombinant MIF-C80W expressed and purified from *E. coli*. Monoclonal antibody 57D9 was generated by immunizing a MIF-knockout mouse^84^ with recombinant MIF expressed and purified from *E. coli*, that was oxidized with hydrogen peroxide (see below for detailed protocol). Immunizations were six times over three months s.c. with 100 μg/100 μL antigen suspended in an equal volume ABM-ZK (Linaris, Germany) adjuvant. The last immunization was the day before termination i. p. Fusion was performed with P3U myeloma cells (Sigma) according to standard protocols.^47^

Single hybridoma cells were seeded into 96-well plates and grown to confluency. The supernatant was then tested by ELISA with coated H_2_O_2_-induced wt-MIF (oxMIF), native wt-MIF, urea-treated denatured wt-MIF, or BSA as negative control. The isotype of the antibodies was determined with the IsoStrip Mouse Monoclonal Antibody Isotyping Kit (Roche).

#### Purification of mAb 17F3

50 mL of PHFM-supernatant containing secreted mAb17F3 were dialyzed twice against 20 mM TRIS pH9 and then filtered through a 0.45 μm syringe filter. The filtrate was then loaded on a 1 mL Q-sepharose column. The column was washed with 3 mL of 20 mM TRIS pH9 and bound antibody was eluted by addition of PBS. The eluate was concentrated and the buffer changed to PBS using an Amicon filter cartridge with a cut-off of 10 kDa.

#### Epitope mapping of monoclonal antibodies

For epitope mapping of mAb17F3 and imalumab we purchased a PepSpots peptide array from JPT. Each spot of the array carried appr. 5 nmol of peptides covalently bound to a cellulose-β-alanine membrane. In total, the membrane presented 52 overlapping peptides, each with 13 amino acids, covering the complete MIF protein. The membrane was stored at −20°C until use. Epitope mapping was performed according to the manufacturer’s recommendations. Briefly, the membrane was rinsed in a small volume of MeOH, washed with TBS-T for 3 × 3 min and then blocked with 5% milk in TBS-T over night. At the next day the membrane was incubated with a 1:100 dilution of the 17F3 hybridoma supernatant or a 1:1,000 dilution of imalumab for 3h. Then the membrane was washed 3 × 5 min with TBS-T und incubated with fluorescent secondary antibody (Anti-Mouse IgG – IRDye 800CW; Li-COR Biosciences.) diluted 1:20,000 in TBS-T. After washing with TBS-T (3 × 5 min) the membrane was scanned with an LI-COR Odyssey CLX. A secondary only control was carried out before the membrane was probed with the primary antibodies. Between each experiment the membrane was regenerated according to the manufacturer's recommendations.

#### Generation of recombinant vectors

For the generation of a tau-P301S expression plasmid the multiple cloning site (MCS) of the pLHCX vector (TaKaRa) was modified by inserting a synthetic MCS-sequence. For this the modified_MCS_fw and modified_MCS_rev oligonucleotides were annealed, cut with FastDigest HindIII and ClaI (both Thermo Fisher Scientific), and then inserted into pLHCX that was opened with HindIII and ClaI and dephophorylized with FastAP (Thermo Fisher Scientific). The ligation product (pLHCX-mod-MCS) was transformed and amplified in DH5α (Thermo Fisher Scientific). The open reading frame (ORF) of human Tau 2N4R including the complete 3′-UTR was ligated into pLHCX-mod-MCS via XhoI and BamHI. Then the P301S mutation was introduced by site-directed mutagenesis (CCG>TCG) using the QuikChange Site-directed Mutagenesis Kit (Stratagene) and the oligonucleotides Tau5 P301S FOR and Tau6 P301S REV.

For the generation of MIF expression constructs the ORF of human wildtype MIF was amplified by PCR from the vector pCMV6-entry MIF (Origene) and ligated into the pET11a vector (Novagen) via NdeI/BamHI using the pET11a MIF-fw and pET11a MIF-rev oligonucleotides allowing the tag-free expression in *E.coli*. The C80W-mutation was inserted into pET11a-hMIF using the Quik change site directed mutagenesis kit (Stratagene) and the MIF-C80W_fw and MIF-C80W_rev oligonucleotides. For generation of MIF-knockdown rescue constructs the ORF of MIF-wt and MIF-C80W were amplified from pET11a-hMIF or pET11a-hMIF-C80W with the oligonucleotides MIF_HindIII_fw and MIF_ClaI_rev. The purified PCR products were then ligated into pLNCX2 (TaKaRa). To allow expression in MIF-knockdown cells, rescue mutations were inserted by *in vitro* mutagenesis using the oligonucleotides MIF-ko-rescue-fw and MIF-ko-rescue-rev.

#### Generation of retroviruses

Retroviruses were produced in GP2-293 cells (Clontech). A confluent 10 cm dish with GP2-293 cells were split 1:4 and seeded into T75 cell culture flasks that were coated with Poly-Ornithine (Sigma). At the next day, when the cells reached about 70% confluency, the cells were transfected with 9.75 μg pLHCX-tau-P301S, 9.75 μg pVSV-G (Addgene) and 58.5 μL Gene Juice diluted in Optimem (Gibco) following preincubation for 20 min. After changing the medium of the GP2-293 cells the Gene Juice:DNA complex was added to the cells in a drop-wise manner. The cells were incubated for 48h. Then the medium was carefully removed and replaced with 6.5 mL of fresh medium. The retroviral particles were collected after 24h, passed through a 0.45 μm filter (VWR) and aliquots were stored at −20°C until use.

#### Generation of stable cell lines

SH-SY5Y cells constitutively expressing human tau 2N4R-P301S were generated by retroviral infection with pLHCX-mod-MCS-tau-P301S. 1x10^6^ SH-SY5Y cells were seeded on 6-well plates. 1 mL of the retroviral supernatant was quickly thawed in a 37°C water bath and mixed with 1 mL of fresh DMEM/F12 medium and 10 μg polybrene (Sigma). After incubation of 24h cells were washed with PBS and fresh medium was added. At the next day cells were splitted 1:10 into a T75 flask containing DMEM/F12 and 400 μg/mL hygromycin B (Thermo Fisher Scientific). The selection medium was changed every 2–3 days until stable cell clones appeared.

#### Generation of MIF- and CUTA-knockout lines by CRISPR-Cas9

For generation of the HEK293-MIF-ko as well as SH-SY5Y-tau-P301S-MIF- and -CUTA-ko lines, we identified sgRNA sequences specific for human MIF (exon 1) or human CUTA (exon 1) using CRISPOR.org (https://crispor.org): AGCTCGGAGAGGAACCCGTC (MIF) and CCTTGGCGACCTT CTCGTTG (CUTA). As negative control we used a scrambled sgRNA sequence: GCACTCACATCGCTACATCA (Applied Biological Materials, Richmond, BC, Canada). MIF-ko-fw, MIF-ko-rev, CUTA-ko-fw, CUTA-ko-rev, as well as CRISPRctrl_fw, and CRISPRctrl_rev were phosphorylated with T4 PNK (Thermo Fisher Scientific) in T4 ligation buffer (Thermo Fisher Scientific). After annealing each set of oligonucleotides, they were ligated into the lentiCRISPRv2 shuttle vector (Addgene) that was linearized with BsmBI (NEB) and dephosphorylated with FastAP (Thermo Fisher Scientific). Finally, the product was transformed into Stbl3 bacteria (Invitrogen). The cloning products were validated by sequencing.

Lentiviral particles were produced in 90–100% confluent HEK293-FT cells (Clontech) using a T75 flask. The cells were washed once with PBS and 5 mL Optimem (Invitrogen) were added 1h before transfection. The transfection mixture was prepared in 667 μL Optimem and contained 7 μg of the lentiviral shuttle vector as well as 4 μg pMDLG, 4 μg pRSV_Rev, and 4 μg pMD2.G (all Addgene) for packaging. In another tube, 640 μL Optimem were supplemented with 27 μL Lipofectamine 2000 (Thermo Fisher Scientific), and both mixtures were incubated for 5 min at RT. The DNA was then combined with the Lipofectamine-containing medium, followed by 20 min incubation at RT before it was added to the cells in a dropwise manner. After 14h the medium was changed to 6 mL of normal growth medium (DMEM). Following another 24h of incubation the cell culture supernatant containing the viral particles was passed through a sterile 0.45 μm filter (VWR) to remove cell debris. Aliquots of 1 mL thereof were stored at −20°C.

For infection with lentiviral particles SH-SY5Y-tau-P301S cells were seeded into T25 flasks. At the next day the medium was replaced with 1 mL fresh complete growth medium, 1 mL of the viral particles and 10 μg polybrene (Sigma). One flask was left uninfected for selection control and was provided with 2 mL polybrene-containing growth medium instead. After incubation for 24h the medium was removed and cells were washed twice with PBS. Then 6 mL of fresh growth medium were added and cells incubated for another 24h. For selection the cells were trypsinized and resuspended in 10 mL growth medium containing 1 μg/mL of the selection marker puromycin and transferred to a new T75 flask. The medium was changed every 3–4 days and selection marker was added until stable cell clones appeared and non-infected control cells completely died. The knockdown efficiency of MIF was analyzed by western blot.

#### Transient transfection

Transient transfection of HEK-MIF-knockdown cells with MIF-rescue constructs were carried out with lipofectamine 3000 according to the manufacturer’s recommendations. Briefly, 5x10^5^ HEK-MIF-ko cells were seeded into 12-well plates. At the next day the cells were transfected with 2.5 μg of vector DNA. Cells were washed twice with PBS and lysed in PBS/1% NP40 48h after transfection.

#### HSV-1 KOS viral stock generation

The HSV-1 KOS strain (ATCC-VR-1493) with 2x10^7^ plaque-forming units (pfu)/mL was used to prepare viral stocks after infection of Vero cells (ATCC-CCL-81). A confluent 75-cm^2^ flask with Vero cells was infected with HSV-1 KOS at a multiplicity of infection (MOI) of 0.01 pfu/cell. For this the virus was diluted in 3 mL of Medium 199 (Gibco). After incubation for 2h at 37°C the medium was aspirated and 3 mL of fresh DMEM (Gibco) supplemented with 5% New Born Calf Serum (NBCS; Gibco) was added to the flask. The virus was allowed to replicate for 3 days. Then the virus was released by freeze thawing following 3x sonication for 30 s using a Misonix ultrasonic water bath at 100% amplitude. Aliquots of the virus were stored in liquid N_2_. The virus titer was determined by virus plaque assay.

#### Virus plaque assay

Virus titer determination was carried out in 25-cm^2^ flasks or 6-well plates with confluent Vero cells. The virus stock was serially diluted in Medium 199. 1 mL of each of the dilutions was added to the Vero cells in duplicates and incubated for 2h. Then the inoculum was aspirated and DMEM supplemented with 5% NBCS and 7.5 μg/mL human immunoglobulin (Sigma) was added. After two days the cells were washed twice with PBS and fixed with MeOH for 5 min at RT. Then 1:10 diluted KaryoMax Giemsa stain solution (Gibco) was added to the cells and incubated for 20 min at RT. Following washing with water the plaques were counted under the microscope and the titer was calculated.

#### Infection of cell lines with HSV-1

4x10^5^ Vero cells, 8x10^5^ SH-SY5Y or 2x10^6^ differentiated LUHMES (differentiation day 6) cells were seeded into 12-well plates. After 24h the cells were treated with compounds (as indicated in the figures) and after 1h they were infected with HSV-1 applying MOIs as described in the figures. After 20h (or at indicated time points) cells were either washed twice with PBS, then lysed in cold PBS/1% NP40 including the cOmplete EDTA-free protease inhibitor cocktail (Roche) and phosphatase inhibitor cocktail (Sigma) or virus was released by freeze-thawing and ultrasonic treatment for analysis in viral plaque assay (see above).

#### In-cell ELISA

Antiviral activity of the compounds was measured by in-cell ELISA that was adapted from Fabiani et al.^90^ 4x10^4^ Vero cells were seeded in 96-well plates and grown to confluency for 24h. Then, the medium was removed and cells were treated with serially diluted compounds or DMSO (Sigma) added to 50 μL of Medium 199. Thereafter, the cells were infected with 50 μL of HSV-1 (MOI of 1) diluted in Medium 199. In parallel cells were infected with serial dilutions of HSV-1 to generate a standard curve. After incubation for 2h the wells were washed twice with PBS and then 100 μL DMEM plus compound or DMSO were added and the cells were incubated for 24h at 37°C. At the next day, the cells were washed twice with PBS, fixed with 50 μL of PBS/4% paraformaldehyde (PFA) for 15 min and then permeabilized with 50 μL of PBS/0.1% TX-100 (Sigma) for 5 min at RT. Following blocking with 5% BSA in PBS/0.05% Tween 20 (Sigma, PBS-T) for 60 min the cells were incubated with 50 μL of anti-HSV-1-gC antibody (abcam) diluted 1 to 10,000 in PBS-T over night at 4°C. The following day, cells were washed four times with PBS-T and then incubated with Hrp-conjugated goat anti-mouse antibody (Thermo Fisher Scientific) at a dilution of 1:10,000 in PBS-T for 60 min. Then, cells were washed four times with PBS-T before adding 50 μL of the substrate cocktail (OptEIA, BD). The reaction was stopped after 30 min by adding 50 μL of 2N H_2_SO_4_ and signals were spectrometrically analyzed using a Tecan Safire at 450 nm.

#### Infection of human brain organoids

60 days old human brain organoids with an estimated cell number of 2.5x10^6^ were preincubated with DMSO or 250 nM of PAV-174 for 1h (final DMSO concentration was 0.5%). Then organoids were infected with HSV-1 at an MOI of 0.1 or 1.0. At the next day the medium was renewed and new compound was added. After another 24h, the organoids were washed two times with PBS and then either fixed with 4% PFA at 4°C overnight (for immunocytochemistry and TUNEL assay) or lysed with cold RIPA buffer (50 mM Tris-HCL, pH 7.4, 1% NP-40, 0.5% Na-deoxycholate, 0.1% SDS, 150 mM NaCl, 5 mM EDTA, 50 mM NaF) plus 1X Protease inhibitor cocktail (Roche) and phosphatase inhibitor cocktail (Sigma) followed by 4x sonication for 30s using a Misonix ultrasonic water bath at 100% amplitude. Protein concentration was determined with the DC Protein Assay (Bio-Rad). For immunocytochemistry and TUNEL assay fixed organoids were embedded in paraffin and section of 5 μm were prepared.

#### TUNEL assay

Apoptotic cells in sections of brain organoids were detected by TdT-mediated dUTP-biotin nick end labeling (TUNEL) using the DeadEnd Fluorometric TUNEL System (Promega) according to the manufacturers recommendations. At least three 5-μm sections of the brain organoids were analyzed by taking at least 10 images of each organoid. Dead cells were counted using Fiji/ImageJ (NIH).

#### Immunocytochemistry

For immunocytochemistry analysis 5x10^4^ LUHMES cells at differentiation day 2 were seeded on 13 mm coverslips that were coated with L-ornithine and fibronectin (see above) and placed into 24-well plates. After four additional days of differentiation the cells were infected with HSV-1 (MOI = 1). After 24h the cells were carefully washed with PBS and then fixed with PBS/4% PFA for 15 min at RT. The fixed cells were then permeabilized and blocked with 1%BSA (Sigma)/0.5% saponin (Sigma) and 5% (w/v) nonfat milk (Oxoid) for 1h at RT. Anti-HSV-1-gC diluted 1:500 in 1%BSA/0.5% saponin was applied overnight at 4°C. At the next day the cells were washed three times with PBS and then incubated with anti-mouse Alexa Fluor 488 (Invitrogen) diluted 1:1,000 in PBS for 1h at RT. Following washing three times with PBS and two times with water, cells were mounted with ProLong Gold with DAPI (Invitrogen), and imaged with a Zeiss Axiovision Apotome.2 microscope.

For immunocytochemistry of organoid sections, frozen slides were thawed and kept on RT until they were dry. Slides were washed with PBS and incubated for 5 min. Then the sections were incubated with 30 mM glycine in PBS for 5 min and permeabilized with PBS/0.5% TX100/0.1% Tween 20 for 10 min. To equally distribute the solutions a parafilm layer was placed on top. Following permeabilization the sections were blocked with PBS/0.5% fish gelatin/0.1% TX-100 for 2h at RT. Anti-HSV-1-gC was diluted in blocking solution (1:500) and incubated at 4°C overnight. At the next day the sections were washed three times with blocking solution before adding the secondary antibody (Anti-Mouse IgG-Alexa Fluor 594) diluted 1:500 in blocking solution for 4h at RT. Again, sections were washed three times with blocking solution and the anti-tubulin antibody (TUJ1, 1:500) was added and incubated over night at 4°C. The following day sections were washed three times with blocking solution before they were incubated with anti-Rabbit IgG-Alexa Fluor 647 (1:500) for 4h at RT. Finally, sections were washed two times with PBS and two times with water before mounting with Mowiol (Sigma). Raw data were collected using a Leica SP8 confocal system.

#### Tau assay

For the analysis of compound effects on tau phosphorylation 4x10^5^ SH-SY5Y tau-P301S cells were seeded on 24-well plates. At the next day the cells were treated with compounds diluted in DMSO. Final concentration of DMSO was 0.2%. After an incubation at 37°C for 48h the cells were washed twice with PBS and then lysed in PBS/1% NP40 (Applichem) including the cOmplete protease inhibitor cocktail 2 (Roche) and phosphatase inhibitor cocktail (Sigma). The protein concentration of the samples was determined with the DC Protein Assay (Bio-Rad).

#### Immunoblot

After the protein concentration of cell lysates and homogenates had been determined using the DC Protein Assay Kit (Bio-Rad), 20 μg of each sample were dissolved in NuPAGE LDS Sample buffer (including 2% β-mercaptoethanol), boiled at 100°C for 5 min and then separated on NuPAGE Novex 4–12% Bis-Tris midi gels (Invitrogen) using NuPAGE MES SDS Running buffer (Invitrogen). Proteins were then transferred to a 0.2 μm nitrocellulose membrane (Amersham) in 24 mM Tris/192 mM glycine/20% MeOH at 400 mA for 2h using a tank blot system (Bio-Rad). In order to detect any blotting artifacts, the proteins on the membranes were visualized with Ponceau S (Sigma). The membranes were destained with PBS and then blocked with PBS/5% nonfat milk (Oxoid) for 1h at RT. Incubations with primary antibodies (diluted 1:1,000) in PBS-T were carried out overnight at 4°C. Then the membranes were washed three times with PBS-T for 10 min and afterward incubated with fluorescent secondary antibodies (IR Dye 680 or 800: Li-COR Biosciences.) diluted 1:20,000 in PBS-T for 1h at RT. Following three washing steps with PBS-T for 10 min each, the membranes were scanned using an LI-COR Odyssey CLX. Signal intensities were calculated using the Image Studio Version 2.1 software (LI-COR Biosciences). Blots were reused for staining with additional primary antibodies following the same procedure.

#### Standard ELISA

The binding of antibodies to a native MIF conformer was determined by a standard ELISA procedure. 96-well-Immuno Maxisorp plates (Nunc) were coated with 500 ng of native MIF or H_2_O_2_-induced oxMIF (6 mM H_2_O_2_ at RT for 6h) that were diluted in 50 mM carbonate buffer pH9.4 with or without 8M urea overnight. The wells were blocked with PBS/5% BSA/0.1% Tween 20 for 2h at RT. After washing with PBS-T, primary antibodies were added to the wells and incubated for 4h at RT. Then, the wells were again washed 3x with PBS-T before a goat anti-mouse IgM-HrP conjugate (for detection of 57D9; 1:2,000 dilution) or a goat anti-mouse IgG-HrP conjugate (for detection of 10C3; 1:10,000 dilution) was added for 1h at RT. After 4x washing with PBS-T the ELISA was developed with 1-step Ultra TMB ELISA (Thermo Scientific).

#### Sandwich ELISA

For quantitative determination of oxidized MIF (oxMIF) by imalumab, 96-well-Immuno Maxisorp plates (Nunc) were coated with 0.5 μg/well of 10C3 in 50 mM carbonate buffer pH 9.4 overnight. The wells were blocked with PBS/5% BSA/0.1% Tween 20 for 2h at RT. After washing with PBS-T, samples, either 25 μg brain homogenate or 20 μL of cell culture supernatant were diluted in 100 μL PBS/1% BSA/0.1% Tween 20, were added and incubated overnight at 4°C. The wells were washed 3x with PBS-T and incubated with the oxMIF specific antibody imalumab at a 1:2,000 dilution for 4h at RT. Then, the wells were again washed 3x with PBS-T before a goat anti-human IgG-HrP conjugate (Southern Biotech; 1:5,000 dilution) was added for 1h at RT. After 4x washing with PBS-T the ELISA was developed with 1-step Ultra TMB ELISA (Thermo Scientific). For generation of an oxMIF standard curve, recombinant wt-MIF was treated with 0.2% Proclin300 (Sigma) that transforms recombinant MIF into an oxMIF surrogate as described in.^28^

For quantification of oxMIF by 17F3, 0.5 μg/well of purified 17F3 were coated to the wells and bound MIF was detected by the polyclonal anti-MIF antibody (HPA003868; 1:1,000 dilution) and a goat anti-rabbit IgG-Hrp conjugate (1:7,500 dilution).

#### MTT assay

For viability analysis 4x10^4^ Vero or 8x10^4^ SH-SY5Y-tau-P301S cells were plated in a 96-well plate and incubated at 37°C overnight. Then the compounds diluted in DMSO were added to the cells. The final concentration of DMSO was 1%. The cells were then incubated for 24h (Vero) or 48h (SH-SY5Y). Afterward 20 μL of a Thiazolyl Blue Tetrazolium Bromide (MTT, Sigma) solution (5 mg/mL in PBS) was added to the cells and incubated for 4h allowing viable cells to reduce the yellow MTT into blue formazan metabolites. Following aspiration of the medium, the formazan was resuspended in 200 μL isopropanol/40 mM HCl and incubated for 30 min at RT. The diluted formazan was spectrometrically analyzed using a Tecan Safire Spectrometer at 560 nm and background at 670 nm was subtracted.

#### Drug affinity chromatography (DRAC) and proteomics

Pigs were raised in Struve Labs, Manning, Iowa with proper food and free movement as per their IACUC protocol. They were euthanized and brains were removed immediately and homogenized in PBB containing 10 mM HEPES, 10 mM NaCl, 1 mM Mg, and 0.35% TX-100.

10% human brain homogenates were prepared in cold PBB and aliquots were flash frozen in liquid N_2_. Homogenates were centrifuged at 10,000g for 10 min and supernatants were collected. Cells were harvested by scraping into cold PBS and pelleted at 3,000g for 10 min at 4°C. The pellet was then resuspended in PBB yielding a concentration of approx. 5 mg/mL. The lysates were cleared by centrifugation and the supernatants were flash frozen.

For drug resin affinity chromatography (DRAC), supernatant of cell lysates or brain extracts were supplemented with 1 mM of ribonucleoside triphosphates (ATP, GTP, CTP, UTP; NEB), 10 mM creatine phosphate (Sigma) and 10 μL/mL of a 5 mg/mL stock solution of creatine kinase (Sigma). The samples were then loaded onto 20 μL of compound-coupled resin that were equilibrated with PBB-buffer (plus supplements). After one hour of incubation at 26°C, the beads were washed with PBB (100x bed volume). Bound proteins were then eluted either with 200 μM PAV-645 (pig brain) or 100 μM free compound (human brain samples; cell lysates) in the same buffer used as competitor solution or for stripping 1% SDS or 8M urea (cell lysates) for 1h. All elutions were kept frozen until further analysis.

For mass spectrometric identification, pig brain eluates were run on a freshly prepared 12% SDS gel made up with all filtered solutions and chemicals to avoid Keratin contamination. Proteins bands were separated by gel electrophoresis at 100 V, constant for 1.5h. The marker and protein bands were fixed and stained with freshly prepared Coomassie blue (G250). All stains and destains were carried out with filtered solutions and all operation was done in a laminar flow hood to avoid keratin contamination. Each stained band (as visualized under a white light box) was sliced (1 mm^3^ per well (in 96 well MS/MS reaction plate (Intavis AG) in duplicates and sent out to USDA lab, Richmond, CA for further analysis. They were digested and analyzed by mass spectrometry (With Scaffold-2 viewer).

#### Expression and purification of recombinant MIF

For expression of recombinant human wildtype-MIF and MIF-C80W BL21-(DE3)-Rosetta-pLysS bacteria (Novagen) were transformed with pET11a-MIF or pET11a-MIF-C80W and spread on LB-Agar plates containing 50 μg/mL carbenicillin (Duchefa) and 34 μg/mL chloramphenicol (Sigma). One colony was used to inoculate a preculture of either 10 mL of 2-YT consisting of 16g/L tryptone (Serva), 10 g/L yeast extract (Sigma) and 5 g/L NaCl or 10 mL M9 medium (plus ^13^C -labeled glucose and ^15^N-labeled NH_4_Cl) for expression of ^13^C- and ^15^N-labeled MIF, overnight at 37°C. At the next day 2 × 500 mL 2-YT or 2 × 500 mL M9 medium containing ^15^N-labeled NH_4_Cl and ^13^C labeled glucose were inoculated with the preculture.^83^ The bacteria were grown to an OD_600_ of 0.9 at 37°C before expression of MIF was induced by adding 1 mM IPTG (isopropyl β-D-thiogalactopyranoside; Applichem). MIF was expressed overnight at 18°C. At the next day the bacteria were harvested by centrifugation, resuspended in 20 mM NaPi pH7.0 and lysed by sonication. The lysate was cleared by centrifugation at 20,000g for 30 min and the supernatant was filtered through a 0.45 μm filter (VWR). In order to purify MIF, the lysate was first passed over a 5-mL CM-sepharose column (GE Healthcare), and after washing with 10 column volumes (CV) of 20 mM NaPi pH7.0 MIF was eluted with 20 mM NaPi pH8.0. The eluate was then passed over a 5 mL Q-sepharose column (GE Healthcare). The Flow through was then concentrated using Amicon Ultra-4 centrifugal filters with a cut-off of 10kDa. The purity of MIF was validated by SDS-PAGE and aliquots were stored at −80°C.

#### NMR structure

The NMR F_1_-[^13^C,^15^N]-filtered-[^1^H,^1^H]-NOESY spectra for structure calculation were recorded on a 700 MHz Bruker Avance *Neo* spectrometer equipped with cryoprobe, and the ^15^N-HSQC were recorded on a 600 MHz Bruker Avance III spectrometer equipped with cryoprobe. The filtered NOESY were recorded for MIF 1.0 mM and saturation of PAV-174, i.e., 1.0 mM of PAV-174. at 310K to reduce the rotational correlation time of the system, with two different mixing times 60 and 100ms; the free induction decay was measured for 106ms (2048 points) and the indirect dimension was set to 20ms (400 points); the signal acquisition was performed with 160 scans and the interscan delay was set to 1.5s. The ^15^N-HSQC were measured at 298K with 121ms (2048 points) in the direct dimension and 53 ms (256 points) in the indirect dimension; the signal acquisition was performed through the accumulation of 16 scans and an interscan delay of 1s was applied. The cross-peaks corresponding to ligand-proteins were used to derive ligand-protein distant restraints using the initial rates of the normalized NOE intensity build-up curves.^91^ The intermolecular distance restraints involved four different protein aromatic signals and only one protein methyl, consistently with the binding site suggested by the ^15^N-HSQC spectra, which is highly populated with aromatic residues. The intermolecular distance restraints were kept semiambiguous, i.e., only the ligand signals were assigned, and the MIF- PAV-174 complex was calculated with the NMR^2^ software^92^ using the CYANA software for tortion angle dynamics structure calculation.

A series of eight ^15^N- T_1_-HSQC with varying T_1_ delays of 0, 40, 70, 110, 150, 200, 250, and 300 ms were acquired. The ^15^N- T_1_-HSQC relaxation experiments were measured at 298K with 121ms (2048 points) in the direct dimension and 53ms (256 points) in the indirect dimension; the signal acquisition was performed through the accumulation of 32 scans and an interscan delay of 1.2 s was applied.

A series of eight ^15^N- T_1ρ_-HSQC with varying T_1ρ_ delays of 5, 10, 20, 40, 60, 80, 100, and 120 ms. The ^15^N- T_1_-HSQC relaxation experiments were measured at 298K with 121ms (2048 points) in the direct dimension and 53ms (256 points) in the indirect dimension; the signal acquisition was performed through the accumulation of 64 scans and an interscan delay of 1.5 s was applied.

The relaxation rates were obtained by fitting the signal decays with the software Relax.

#### SEC-HPLC-MALS

The samples for SEC-HPLC-MALS were 100 μM of MIF (WT or mutant C80W) in 20 mM phosphate buffer, with or without compound PAV-174 (100 μM).

The HPLC-MALS was performed at 0.5 mL/min and 25°C with an Agilent 1200 series HPLC system and a size exclusion column TSKgel G2000SWXL from Tosoh bioscience. The detection was performed recording the UV absorbance at 280 nm with the HPLC detector and the light scattering with a miniDawn Treos spectrometer from Wyatt. The molecular weight of the detected species is calculated using the extinction coefficients at 280 nm (12950 M^−1^ cm^−1^ for WT and 18450 M^−1^ cm^−1^ for C80W), absorbance at 280 nm and the Rayleigh ratio using the built-in function in Astra software.

#### Animal experiments

TgTau58/2 mice^44^ were a kind gift from Novartis (Boston, USA). Two months-old males, heterozygous for the tau-P301S transgene (*n* = 12 per group), were either treated with 10% DMSO or 5 mg/kg body weight compound PAV-617 for four weeks administering 100 μL volumes intraperitoneally three times a week, after which animals were perfused with PBS and terminated. The brains were extracted cut into two-halves and flash frozen in liquid N_2_. One brain halve was homogenized in cold extraction buffer (10 mM Tris pH7.4, 1mM EGTA, 0.8M NaCl, 10% sucrose, 1x Protease inhibitor cocktail, 1x phosphatase inhibitor cocktail 2) to 10%. For screening 1% NP40, 0.1mg/mL DNAseI and 5 mM MgSO_4_ were added to the homogenates and after 30 min incubation on ice the protein concentration was determined using the DC Protein Assay Kit (Bio-Rad). Equal amounts of protein were then either directly used for detection of tau in the whole homogenate by immunoblot or sarkosyl insoluble tau was precipitated following a protocol of.^93^ 750 μg lysate were centrifuged at 6,000g for 20 min at 4°C. Then the supernatant was carefully taken and sarkosyl was added to 1% end concentration. After an incubation for 1h at RT the samples were spun down at 100,000g for 1h at 4°C. The supernatants were removed and the pellets carefully washed with 200 μL extraction buffer without sucrose. The pellets were then incubated in 60 μL PBS with 8 M urea at room temperature overnight. 20 μL were used for immunoblot analysis.

### Quantification and statistical analysis

All statistical analyses were performed as indicated using GraphPad Prism (Versions 6 & 9.4); GraphPad Software Inc., San Diego, CA, USA). IC_50_ and CC_50_ values were calculated using the logarithm of inhibitor concentration on GraphPad Prism 6.0. Data are presented as mean ± SEM and appropriate statistical tests and *p*-values are stated in the respective figure legends. Outlier were checked and removed by ROUT analysis (Q = 1%). *p*-values of ∗*p* < 0.05, ∗∗*p* < 0.01, ∗∗∗*p* < 0.001, ∗∗∗∗*p* < 0.0001 were used as significance levels.

## Supplemental information

Document S1. Figures S1–S7

Document S2. Article plus supplemental information
