# Supplementary material for: Oxidized MIF is an Alzheimer’s disease drug target relaying external risk factors to tau pathology
Source: Cell Rep Med. 2025 Dec 18;7(1):102520. doi: 10.1016/j.xcrm.2025.102520 (PMC12866179; doi:10.1016/j.xcrm.2025.102520)
Supplement: Document S1. Figures S1–S7 [file mmc1.pdf]

Cell Reports Medicine, Volume *7*

Supplemental information

Oxidized MIF is an Alzheimer's disease drug target relaying external risk factors to tau pathology

Andreas Müller-Schiffmann, Felix Torres, Anatoliy Kitaygorodskyy, Anand Ramani, Argyro Alatza, Sarah K. Tschirner, Julien Orts, Arthur Haltrich, Ingrid Prikulis, Shaofeng Yu, Debendranath Dey, Suguna Mallesh, Dharma Prasad, Dennis Solas, Verian Bader, Annemieke Rozemuller, Selina Wray, Jay Gopalakrishnan, Roland Riek, Vishwanath R. Lingappa, and Carsten Korth

Supplementary information

Oxidized MIF is an Alzheimer's Disease drug target relaying external risk factors to tau pathology

Andreas Müller-Schiffmann, Felix Torres, Anatoly Kitaygorodskyy, Anand Ramani, Argyro Alatza, Sarah K. Tschirner, Julien Orts, Arthur Haltrich, Ingrid Prikulis, Shaofeng Yu, Debendranath Dey, Suguna Mallesh, Dharma Prasad, Dennis Solas, Verian Bader, Annemieke Rozemuller, Selina Wray, Jay Gopalakrishnan, Roland Riek, Vishwanath R. Lingappa, Carsten Korth

Figure S1

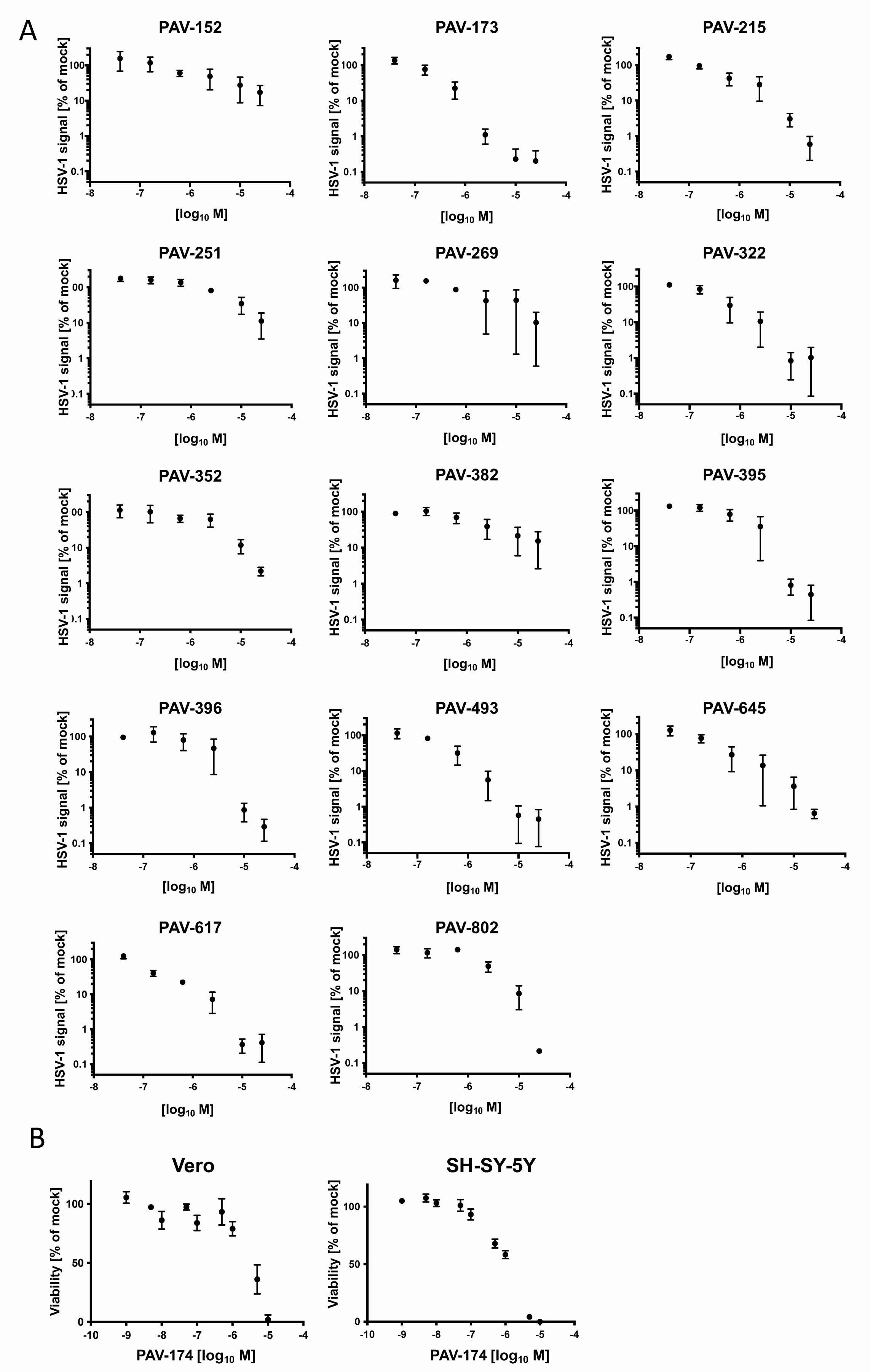
A
PAV-152
PAV-173
PAV-215
PAV-251
PAV-269
PAV-322
PAV-352
PAV-382
PAV-395
PAV-396
PAV-493
PAV-645
PAV-617
PAV-802
HSV-1 signal [% of mock]
[log10 M]
B
Vero
SH-SY-5Y
Viability [% of mock]
PAV-174 [log10 M]

Figure S1. CC_{50} values of compounds, Related to Table 1

(A) The IC_{50} of analogs of PAV-174 were determined by in-Cell ELISA. Each data point displays the mean +/- SEM of three independent experiments (n=3).

(B) MTT assay of PAV-174 in Vero (left) or SH-SY5Y-tau-P301S (right) cells treated with increasing concentrations of PAV-174 resulted in a CC_{50} of 1.12 μM (Vero) or 1.48 μM (SH-SY5Y). Each data point displays the mean +/- SEM of three (Vero) or four (SH-SY5Y) experiments (n=3 or n=4).

Figure S2

A

Protein name	MW (kDa)	Accession number	Peptides identified	Mascot ion score	% coverage
MIF	12	MIF_PIG	(M)PMFVVNTNVPR(A)	51.5	17
			(K)LLCGLLAER(L)	60,9	
CUTA	19	F1RZR6_PIG	(K)GKIEEDSEVLMMIK(T)	37,3	16
			(K)IEEDSEVLMMIK(T)	40,8	
			(K)TQSSLVPALTDFVR(S)	58,7	
GSTP1	23	GSTP1_PIG	(-)PPYTITYFPVR(G)	56,5	27
			(K)FQDGDLTLYQSNAILR(H)	84,8	
			(K)EAALVDMVNDGVEDLR(C)	130,1	
			(K)YATLIYTNYEAGK(E)	41,4	
PRDX2	22	F1SDX9_PIG	(R)NLSLDYGVLKEDEGIAYR(G)	55,5	15
			(R)QITVNDLPVGR(S)	57,2	
PKM2	57	F1SHL7_PIG	(R)NTGIICTIGPASR(S)	56,5	7
			(K)VYVDDGLISLLVK(Q)	61,2	
			(R)GDLGIEIPAEK(V)	52,5	
CALML3	12	F1SQH2_PIG	(K)EAFSLFDK(D)	59,6	42
			(K)EAFSLFDKDGDGTITTK(E)	73,4	
			(K)DTDSEEEIREAFR(V)	46,0	
			(R)VFDKDGNGYISAAELR(H)	77,3	
			(R)VFDKDGNGYISAAELR(H)	69,4	
			(K)DGNGYISAAELR(H)	63,7	
HSPCB	83	F1RQU2_PIG	(R)TLTLVDTGIGMTK(A)	70,3	5,4
			(K)ADLINNLGTIAK(S)	80,6	
			(R)GVVDSEDLPLNISR(E)	69,8	

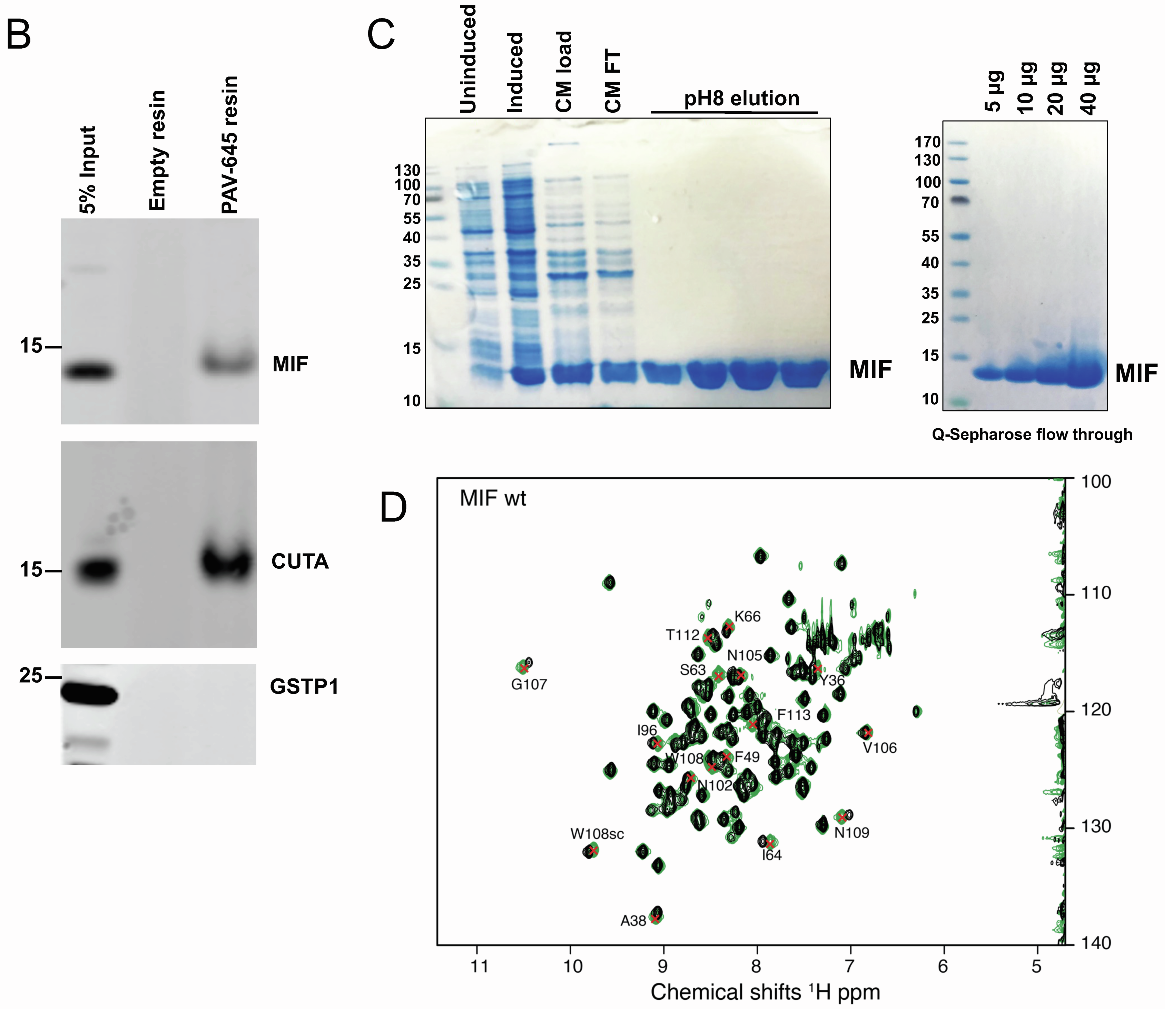

Figure S2. PAV-174 binds MIF at the multimerization interface. Related to Figure 2

(A) List of peptides identified by DRAC analysis including Mascot ion score.

(B) DRAC assay with cell lysates derived from SH-SY5Y-tau-P301S cells that were applied either on empty control resin or resin presenting PAV-645. In contrast to GSTP1, MIF and CUTA were eluted from drug resins by urea. On the left of each blot 5% of input material is shown.

(C) Recombinant expression and purification of human wildtype MIF in *E.coli*. Expression of tag-free MIF was induced in BL21 bacteria and MIF was then purified by ion exchange chromatography (CM Sepharose). The purification steps are shown in the SDS-PAGE on the left. The right image shows the purity (>95%) of concentrated MIF after polishing via Q-sepharose.

(D) ^{15}N-HSQC spectra of the apo-MIF (green) and of the MIF in the presence of PAV-174 (black). The resonances showing the highest perturbations are zoomed in within the squares in **Figure 2B**. The residues showing the most important chemical shift perturbations are shown in the MIF structure in the **Figure 2B**.

Figure S3

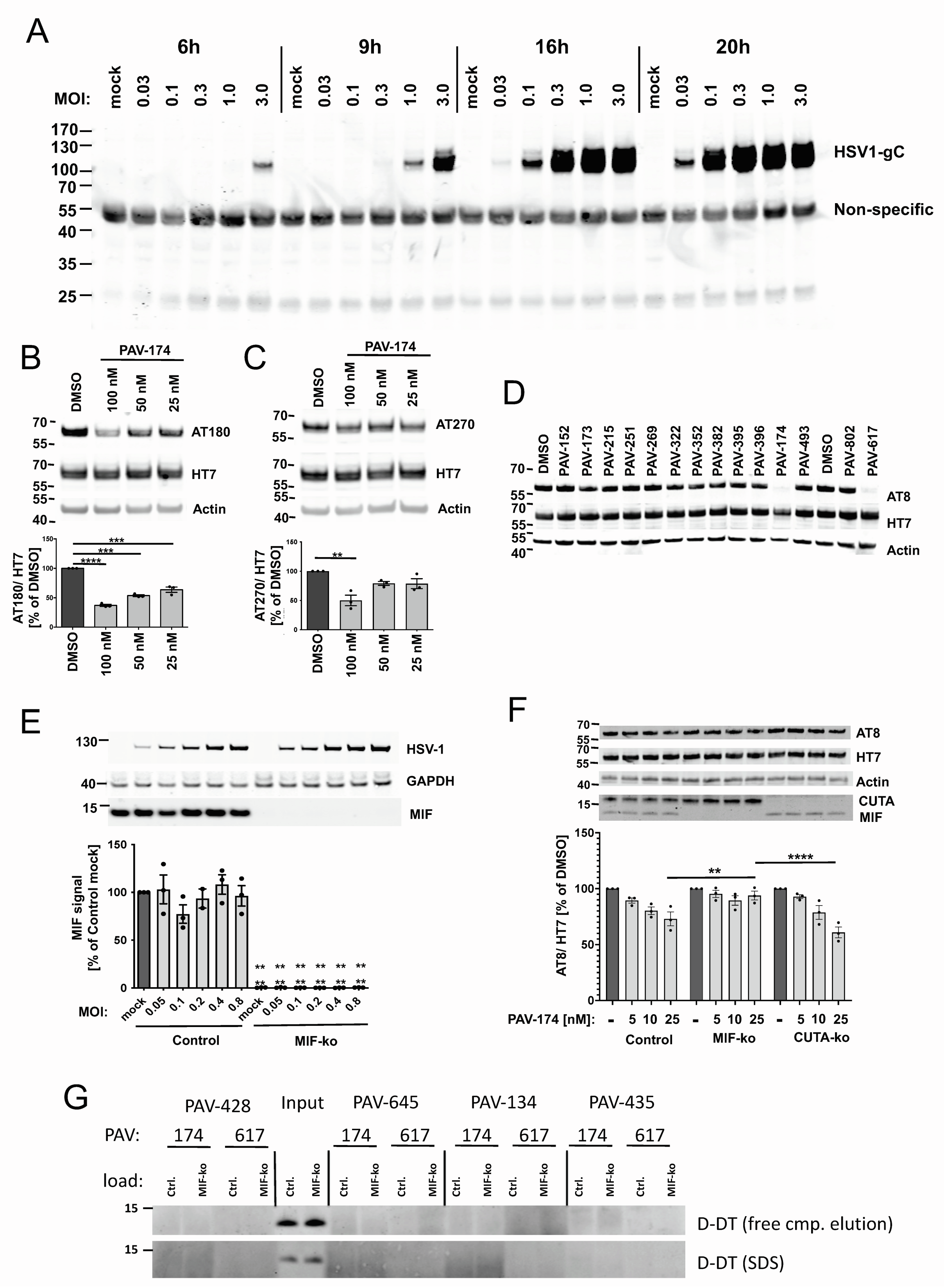

A
6h
9h
16h
20h
MOI:
mock
0.03
0.1
0.3
1.0
3.0
HSV1-gC
Non-specific
B
PAV-174
DMSO
100 nM
50 nM
25 nM
AT180
HT7
Actin
AT180/ HT7 [% of DMSO]
C
AT270
AT270/ HT7 [% of DMSO]
D
AT8
E
HSV-1
GAPDH
MIF
MIF signal [% of Control mock]
Control
MIF-ko
F
CUTA
PAV-174 [nM]:
AT8/ HT7 [% of DMSO]
CUTA-ko
G
PAV-428
Input
PAV-645
PAV-134
PAV-435
PAV:
174
617
load:
Ctrl.
MIF-ko
D-DT (free cmp. elution)
D-DT (SDS)

Figure S3. PAV-174 reduces tau phosphorylation also in the absence of HSV-1-infection in a MIF and dose dependent manner. Related to Figure 3

(A) SH-SY5Y-tau-P301S cells could efficiently been infected with HSV-1. The cells were infected with increasing MOIs (0.03 to 3.0) of HSV-1 and then lysed after 6h, 9h, 16h or 24h. The lysates were analyzed by Western Blot using an antibody against the glycoprotein C (gC) of HSV-1.

(B/C) PAV-174 selectively reduced tau phosphorylation. SH-SY5Y-tau-P301S cells were treated with PAV-174. PAV-174 efficiently reduced tau phosphorylation at Thr231 recognized by AT180 **(B)** but only at high concentrations at Thr181 recognized by AT270 **(C)**. Actin served as loading control. The diagrams show the signals of the phospho-tau specific antibodies normalized to total tau (HT7) from three (n=3) independent experiments. One-way ANOVA (Dunnet´s post-hoc)

(D) Representative Western Blot of results shown in **Figure. 3C**.

(E) Expression of MIF was not modulated upon infection with HSV-1. SH-SY5Y-tau-P301S-CRISPR-control and SH-SY5Y-tau P301S-MIF-ko cells were infected with HSV-1 for 16h and lysates were analyzed for MIF expression. The expression of HSV-1 antigen is shown on top and GAPDH served as loading control. The diagram displays the quantification of MIF normalized to GAPDH derived from three independent experiments (n=3). Two-way ANOVA (Sidak´s post-hoc)

(F) CUTA expression in SH-SY5Y-tau-P301S cells is not necessary for PAV-174 mediated reduction of tau phosphorylation. SH-SY5Y-tau-P301S-CRISPR-control, -MIF-knockdown and CUTA-knockdown cells were treated with the indicated concentrations of PAV-174. The knockdown of MIF and CUTA was verified by Western Blot. PAV-174 dose dependently reduced tau phosphorylation in the control and CUTA-knockdown cell lines but not in the MIF-knockdown line. Data from three independent experiments (n=3) were analyzed. One-way ANOVA (Sidak´s post-hoc)

(G) The MIF homolog D-DT binds not to PAV-617. PAV-617 was either coupled via its via its pyrrolidine ring (PAV-428) or its phenothiazine moiety (PAV-435). As controls PAV-645 resin or empty resin (PAV-134) were used. Lysates derived from SH-SY5Y-tau-P301S control and – MIF-ko cells were loaded on the resins. Elution with free compound and stripping of the resins with SDS revealed no binding of D-DT to PAV-617 with any resin.

Data represents the mean +/- SEM. $^{**}p < 0.01$; $^{***}p < 0.001$; $^{****}p < 0.0001$

Figure S4

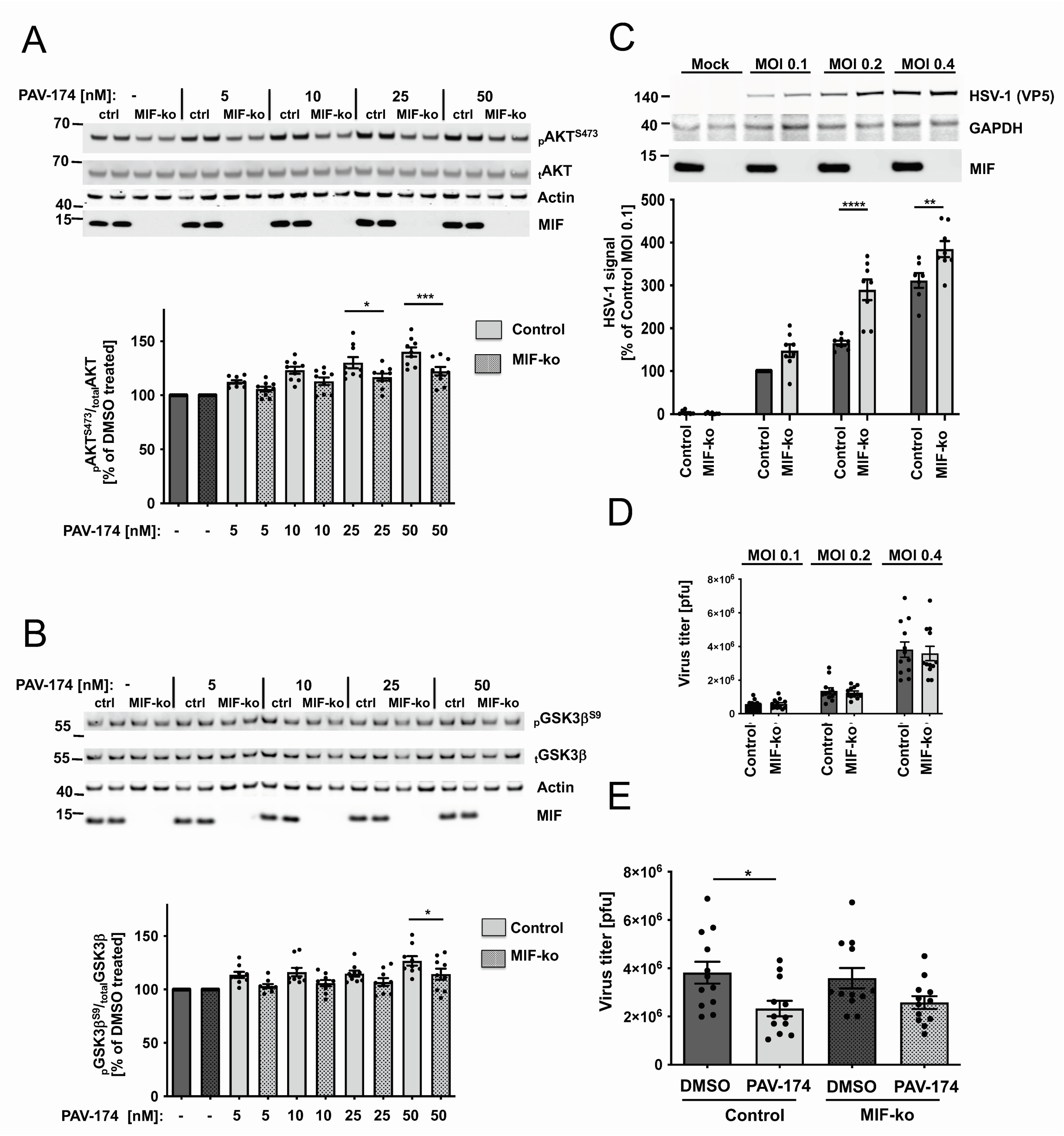

Figure S4. PAV-174 reduces tau phosphorylation via modulation of Akt/GSK3β phosphorylation and HSV-1 replication in a MIF dependent manner. Related to Figure 3

(A/B) PAV-174 induces phosphorylation of Akt at S^{473} **(A)** and GSK3β at S^{9} **(B)** in a MIF dependent manner. SH-tauP301S-CRISPR-control or -MIF-ko cells were treated with increasing concentrations of PAV-174 for 6h. PAV-174 led to a dose dependent and significantly higher phosphorylation of Akt^{473} in SH-SY5Y-tau-P301S-control than in -MIF-ko cells. Similar PAV-174 significantly increased phosphorylation of GSK3β at Ser^{9} already at low concentrations only in SH-tauP301-control but not in -MIF-ko cells. The diagrams show the average values of $pAkt^{S473}$ normalized to total Akt or $pGSK3\beta^{S9}$ normalized to total GSK3β as

% of DMSO treated cells of the respective cell line derived from nine independent experiments (n=9). Two-way ANOVA (Sidak´s post-hoc)

(C) Increased production of HSV-1 capsid proteins in MIF-ko cells compared to control cells. SH-SY5Y-tau-P301S-CRISPR-control and -MIF-ko cells were infected with the indicated MOIs of HSV-1. The amount of viral capsid proteins within the lysate 16h p.i. were determined by Western Blot using an antibody against the VP5 capsid protein of HSV-1. GAPDH was used as internal control. The absence of MIF in SH-tau-MIF-ko was verified using a polyclonal antibody against MIF. The diagram shows the result from eight infections (n=8). Data were normalized to Control (MOI = 0.1) and analyzed by Two-way ANOVA (Sidak´s post-hoc).

(D) Infection of SH-SY5Ytau-P301S-MIF-ko cells led not to a reduced production of HSV-1 infectious particles. SH-SY5Y-tau-P301S control and -MIF-ko cells were infected with the indicated MOIs of HSV-1. The viral titer was determined by plaque assay 16h pi. The diagram shows the result from twelve infections (n=12). Data were analyzed by Two-way ANOVA (Sidak´s post-hoc). No significant differences were found between both cell lines.

(E) MIF is a functional target of PAV-174. SH-SY5Y-tau-P301S-CRISPR control and -MIF-ko cells were infected with HSV-1 (MOI 0.4) and either treated with DMSO or PAV-174 (5 nM). Plaque assays were performed 16h p.i. The diagram shows the viral titers from twelve (n=12) infections and a statistically significant effect on viral titers when PAV-174 was applied to MIF-containing cells. Two-way ANOVA (Sidak´s post-hoc)

Data represents the mean +/- SEM. *p < 0.05; **p < 0.01; ***p < 0.001; ****p < 0.0001

Figure S5.

A

	PAV-174	PAV-617
MTD (mg/kg)	0.5	20
Protein binding (%)	99.9	91.2

B

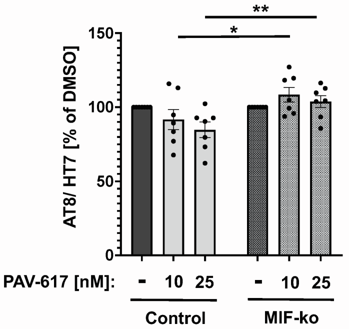

C

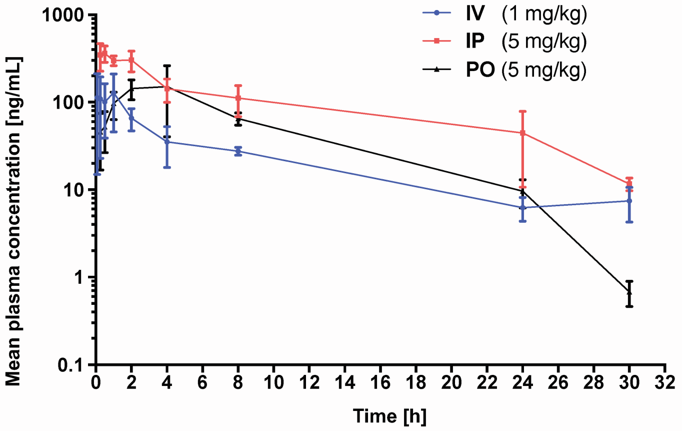

Route	Dose (mg/kg)	AUC (last) (ng.h/mL)	AUC (0 to inf) (ng.h/mL)	Cmax (ng/ml)	Tmax (h)	Kel (1/h)	$t_{1/2}$ (h)	MRT (h)	CL (mL/min/kg)	Vz (L/Kg)	% F
IV	1	747.2	831.5	128.0	1.0	0.1	7.8	7.1	20.0	12.7	
IP	5	2974.7	3093.0	362.6	0.5	0.1	7.0	8.3			80
PO	5	1531.8	1535.1	151.1	4.0	0.2	3.3	6.6			41

D

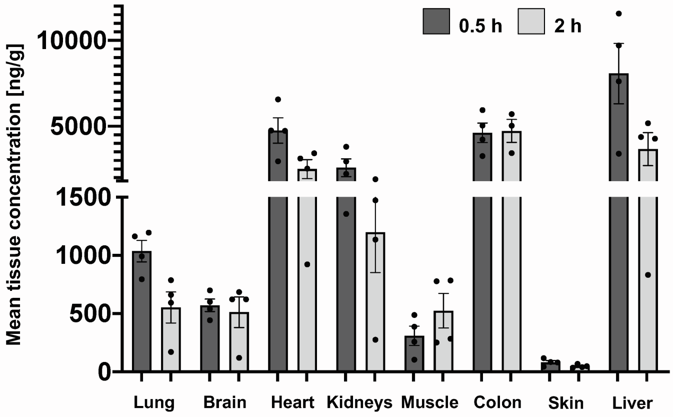

Tissue	Mean tissue Conc (ng/g)	
	0.5 h	2 h
Plasma (ng/mL)	311.4 ± 8.22	287.7 ± 134.0
Lung	1036.5 ± 184.0	553.1 ± 267.3
Brain	570.8 ± 107.6	643.3 ± 36.4
Heart	4752.1 ± 1476.7	2498.1 ± 1114.0
Kidneys	2580.8 ± 1040.2	1197.2 ± 690.0
Muscle	309.4 ± 166.2	524.4 ± 296.7
Colon	4618.3 ± 1144.1	4727.4 ± 233.8
Skin	82.0 ± 30.1	48.5 ± 14.3
Liver	8070.6 ± 3511.7	3664.0 ± 1930.9

Tissue	Tissue/plasma Ratio (ng/g)/(ng/mL)	
	0.5 h	2 h
Lung	3.4 ± 0.7	1.9 ± 0.2
Brain	1.7 ± 0.2	1.8 ± 0.1
Heart	13.3 ± 3.3	9.0 ± 1.1
Kidneys	7.9 ± 4.0	4.0 ± 1.1
Muscle	0.8 ± 0.4	2.1 ± 1.0
Colon	14.3 ± 4.0	13.2 ± 2.4
Skin	0.2 ± 0.1	0.2 ± 0.1
Liver	22.1 ± 10.2	12.2 ± 2.8

E

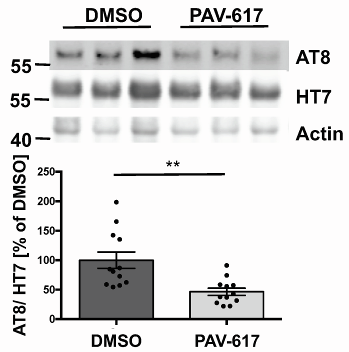

F

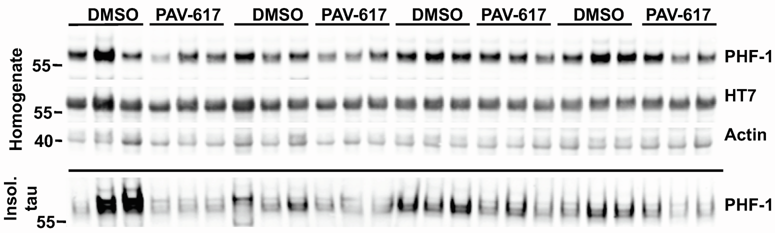

Figure S5. PAV-174 decreases tau phosphorylation and aggregation *in vivo*. Related to Figure 4

(A) MTD (Maximum Tolerated Dose) assessment of PAV compounds in BALB/c mice. BALB/c mice were dosed through intraperitoneal (IP) route at different doses as per body weight of animals and mice were observed at different time interval for toxic signs till 48 h, PD (Post dose). MTD at was declared at NOAEL (No-observed-adverse-effect level) conc. of PAV compound. PAV-617 showed a markedly reduced protein binding (91.2%) compared to PAV-174 (99.9%).

(B) PAV-617 dose- and MIF-dependently reduces tau phosphorylation (AT8). SH-SY5Y-P301S-control or -MIF-knockdown cells were treated with the indicated concentrations of PAV-617 for 48h. PAV-617 reduced tau phosphorylation only in control but not in MIF-knockdown cells. The diagrams show the signals of AT8 normalized to total tau (HT7) from six (n=6) independent experiments. One-way ANOVA (Sidak´s post-hoc)

(C) Pharmacokinetic assessment of PAV-617 in Sprague Dawley rats (SD). Male SD rats (n=4) were dosed via three routes namely intravenous (IV)- 1 mg/kg, (IP)- 5 mg/kg and per oral (PO)- 5 mg/kg. Plasma concentration was analyzed at different time intervals. Pharmacokinetic parameters were determined using WinNonlin. Cmax (peak plasma concentration) achieved through IV was 128 ng/mL, IP was 362.6 ng/mL and PO was 151.1 ng/mL at Tmax (time of peak concentration observed) 1 h, 0.5 h and 4 h respectively. Compound PAV-617 has moderate t1/2 (terminal half-life) through IV- 7.8 h, IP- 7 h and PO- 3.3 h. CL (steady-state clearance) was found to be moderate- 20 mL/min/kg which was 2.75 times lower than rat liver blood flow. Vz (volume of distribution) was found to be high- 12.7 L/kg and F (fraction bioavailability) was found to be very good through IP- 80 % and moderate through PO- 41%.

(D) Tissue partitioning of PAV-617 in SD rats. Male SD rats (n=4) were dosed via IP -5 mg/kg. Plasma and major organs such as lung, brain, heart, kidney, muscle, colon, skin, and liver were collected at 0.5 h and 2 h post dosing. Partitioning of PAV-617 by each organ was analyzed.

(E) Reduction of tau phosphorylation was observed *in vivo* after treating tau58/2 mice with 5 mg/kg of PAV-617. Phosphorylated tau (AT8) was reduced in the homogenates of the treated mice. The diagrams show the average signals of 12 mice per treatment group (n = 12) derived from two independent Western Blots. Data were analyzed by unpaired two-tailed t-test.

(F) Representative blots of results described in **Figure 4B/C**.

Data represents the mean +/- SEM. $*p < 0.05$; $**p < 0.01$

Figure S6.

A

sample	sex	age	braak	amyloid	pmd [h]	ph	weight	apoe
AD 1	f	72	6	C	5,92	6,54	960	32
AD 2	f	68	6	C	3,83	6,50	985	32
AD 3	f	84	6	C	4,83	6,06	960	33
AD 4	f	86	6	C	5,25	6,20	1090	33
AD 5	m	87	5	C	6,17	6,14	1047	33
AD 6	f	90	6	C	4,75	6,39	1045	33
AD 7	m	71	6	C	6,58	6,20	1092	33
AD 8	m	70	6	C	6,33	6,40	1261	43
AD 9	f	89	6	C	4	6,40	977	43
AD 10	m	81	6	C	7,83	6,15	1120	43
N 1	f	60	0	O	8,17	6,58	1310	32
N 2	f	75	1	A	9,17	6,57	1305	32
N 3	f	72	1	A	6,83	7,22	1165	33
N 4	f	78	1	A	7,17	6,32	1120	33
N 5	m	90	2	O	5,75	6,35	1215	33
N 6	m	73	2	A	8	5,37	1553	33
N 7	f	88	3	A	5,42	6,33	1010	33
N 8	f	91	3	A	4,17	6,58	1052	43
N 9	m	83	1	B	5,75	6,35	1590	43
N 10	m	102	3	A	5	6,64	1124	43

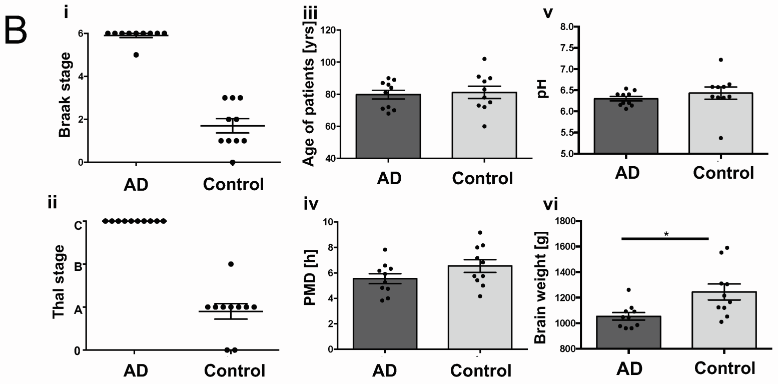

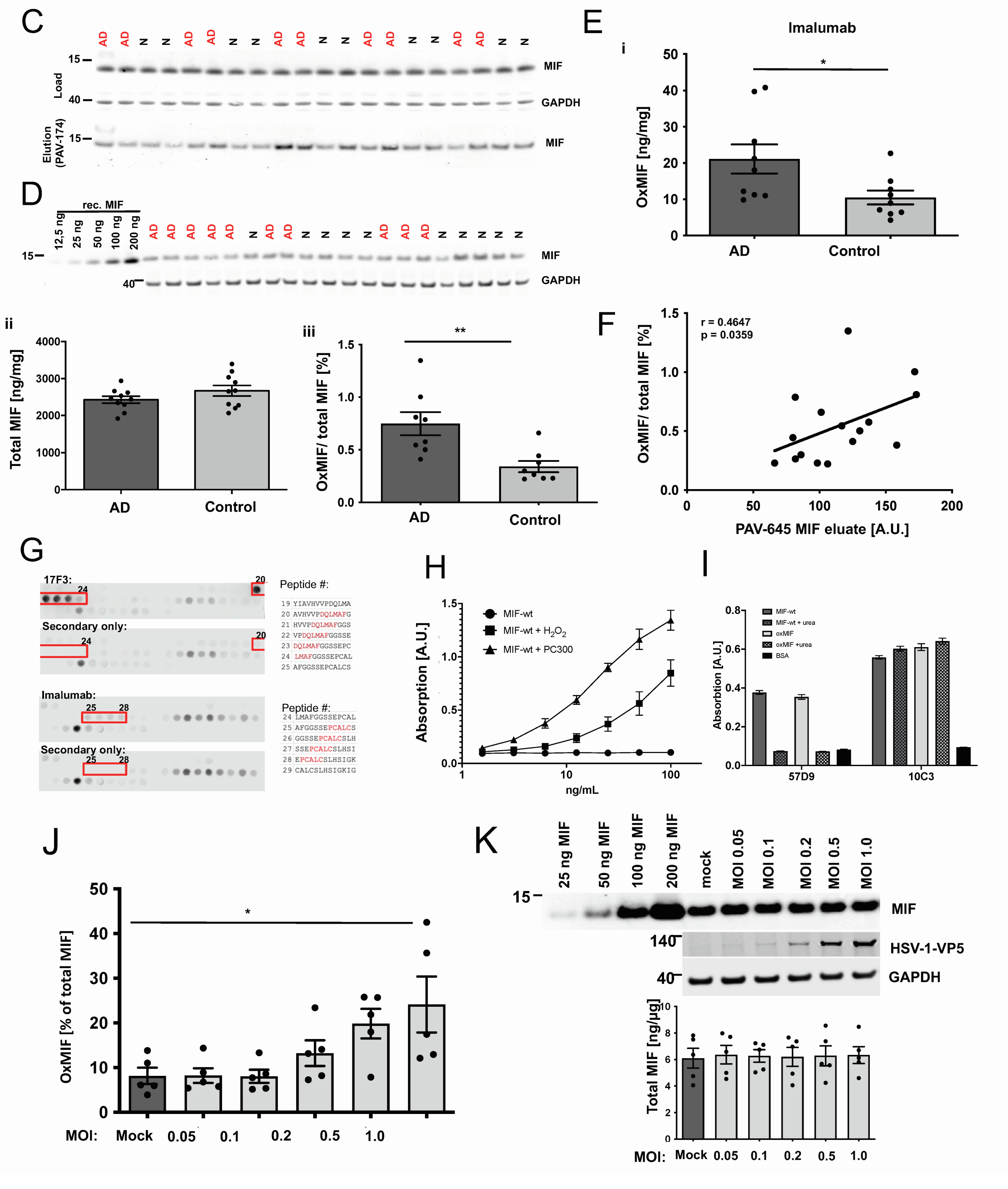

Figure S6. The oxMIF conformer is elevated in *post mortem* brains of patients with Alzheimer's disease. Related to Figure 5

(A) Table describing the brain samples retrieved from the The Netherlands Brain Bank regarding, sex, age, tau pathology (Braak stages), amyloid pathology (according to Thal staging), *post mortem* time (pmd), pH of sample, brain weight and ApoE gene status.

(B) All AD samples displayed severe tau (i) as well as amyloid pathology (ii) and show an equal distribution of age (iii) and no differences in *post mor*tem delay (pmd) (iv) or pH (v). The weight of the brains from the AD patients was significantly reduced compared to the controls (vi). Data of 10 samples per group were analyzed by unpaired two-tailed t-test.

(C) Representative complete Western Blot of DRAC analysis of brain samples shown in **Figure 5A)**. The upper panel displays the loading controls (MIF and GAPDH) of brain homogenates and the lower panel the precipitated and PAV-174-eluted MIF.

(D) Representative quantitative Western Blot of brain samples used for normalizing the oxMIF concentrations determined by the sandwich ELISA using Imalumab shown in **(E)**. Increasing concentrations (12.5 ng to 200 ng) of recombinant expressed human wt-MIF were used to generate a standard curve allowing the quantification of MIF within the brain samples. MIF signals were normalized to GAPDH.

(E) Increased amounts of oxMIF in AD brain samples were detected by Sandwich ELISA using Imalumab. A significant higher oxMIF concentration (ng/mg protein) was detected in AD-brain tissue (i), whereas the total MIF concentration as measured by quantitative Western Blot did not differ (ii). The ratio of oxMIF/ total MIF is shown in (iii). Each data point represents the average of two independent brain sample preparations. Unpaired two tailed t-test

(F) Correlation analysis of the 20 brain samples between oxMIF (% of total MIF) levels detected by Imalumab and the amount of MIF species eluted from PAV-645 resin. The oxMIF levels positively correlated with the amounts of MIF species eluted from PAV-645 resin. Spearman´s rho coefficient and p value (one-tailed) are indicated in the graph.

(G) Linear epitope mapping of 17F3. A filter containing 52 overlapping peptides out of 13 amino acids covering the complete MIF sequence were probed with 17F3, Imalumab or secondary antibody only. Peptides that gave positive reactions not observed with the secondary only control defined the binding epitope (red rectangle). 17F3 binds to a region of MIF (^{45}DQLMAF^{50}) that differs from that of Imalumab (^{56}PCALC^{60}).

(H) 17F3 specifically binds to oxidized MIF-species. In a sandwich ELISA, 17F3 was coated. Increasing concentrations of native MIF as well as H_2O_2- and Proclin300-treated MIF were added as ligands. Bound MIF was detected using a rabbit total MIF antibody. Only H_2O_2- and Proclin300-induced oxMIF were recognized by 17F3.

(I) H_2O_2-induced recombinant oxMIF maintains a native-like conformation. In contrast to 10C3, that recognize a linear epitope within MIF, the conformation-sensitive antibody 57D9 detected

recombinant native MIF as well as H_2O_2-induced oxMIF, but failed to bind to urea-denatured MIF or oxMIF. Data from three (n=3) independent experiments are shown.

(J) Induction of oxMIF in differentiated LUHMES cells upon infection with HSV-1. Differentiated LUHMES cells were infected with increasing MOIs of HSV-1. Cells were lysed 16h p.i and the amount of oxMIF was measured by sandwich ELISA using Imalumab. The results were normalized to total MIF as determined by Western Blot **(K)**. The diagram shows the results from five independent infections (n=5). One-way ANOVA (Dunnet´s post-hoc)

(K) Quantification of total MIF in lysates of differentiated LUHMES cells. Different amounts of recombinant MIF were used to generate a standard curve. MIF signals were normalized to GAPDH. The infection of LUHMES cells with HSV-1 was demonstrated with an antibody against a capsid protein of HSV-1 (VP5). The diagram shows the results from five independent infections (n=5). Data were analyzed by One-way ANOVA (Dunnet´s post-hoc).

Data represents the mean +/- SEM. *$p < 0.05$; **$p < 0.01$

Figure S7

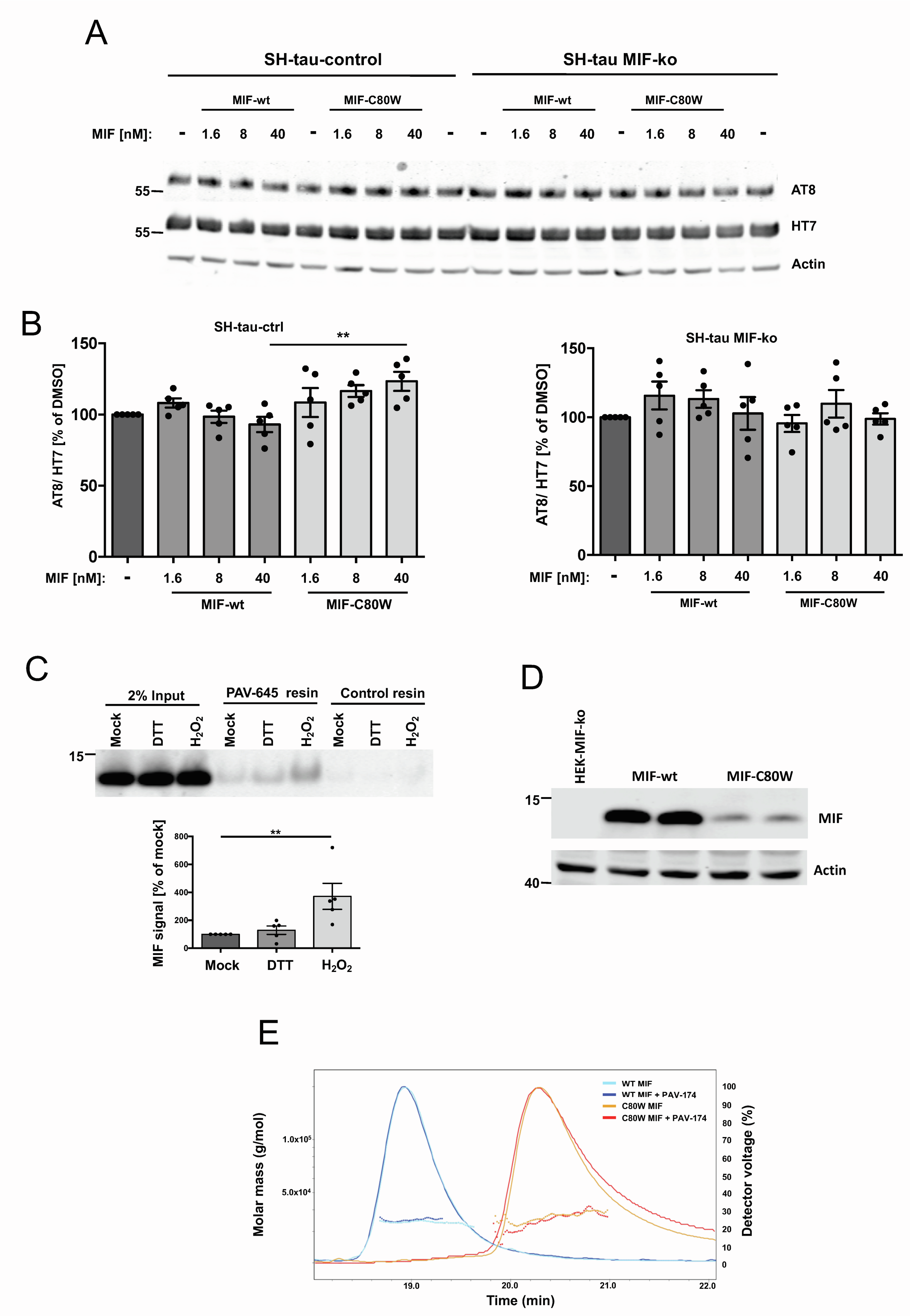
A
SH-tau-control
SH-tau MIF-ko
MIF-wt
MIF-C80W
MIF [nM]: - 1.6 8 40 - 1.6 8 40 - - 1.6 8 40 - 1.6 8 40 -
55
AT8
HT7
Actin
B
SH-tau-ctrl
SH-tau MIF-ko
AT8/ HT7 [% of DMSO]
**
MIF [nM]: - 1.6 8 40 1.6 8 40
MIF-wt
MIF-C80W
C
2% Input
PAV-645 resin
Control resin
Mock DTT H2O2
15
MIF signal [% of mock]
Mock DTT H2O2
D
HEK-MIF-ko
MIF-wt
MIF-C80W
MIF
Actin
15
40
E
WT MIF
WT MIF + PAV-174
C80W MIF
C80W MIF + PAV-174
Molar mass (g/mol)
Detector voltage (%)
Time (min)

Figure S7. OxMIF is a direct driver of tau phosphorylation modulated by PAV-174. Related to Figure 6

(A) Representative complete Western Blot of the analysis presented in Figure 6A. The upper panel displays AT8 and below total tau as detected by HT7 is shown. Actin served as loading control.

(B) Exogenous applied oxMIF induce tau phosphorylation. SH-SY5Y-tau-P301S-control and –MIF-ko cells were treated with recombinant wt-MIF or MIF-C80W for 6h. MIF-C80W dose dependently induced tau phosphorylation in control cells but not in MIF-ko cells. The diagram shows the average values of five independent experiments (n=5). Data were analyzed by One-way ANOVA (Sidak´s post-hoc).

(C) DRAC analysis with reduced (6 mM DTT) or oxidized (6 mM H_2O_2) MIF. Significantly more MIF bound to the PAV-645 resin when MIF was oxidized with H_2O_2. The diagram shows the signal of eluted MIF normalized to non-treated wt-MIF derived from five (n=5) pull-downs. Data were analyzed by One-way ANOVA (Dunnett´s post-hoc).

(D) Transient expression of recombinant MIF-wt rescue and MIF-C80W-rescue in HEK-MIF-ko cells revealed lower levels of MIF-C80W. HEK-MIF-ko cells were transfected with pLNCX-MIF-wt-rescue or pLNCX-MIF-C80W-rescue. After 48h cells were harvested and lysates were subjected to Western Blot. Actin was used as loading control.

(E) Chromatograms of the different sample injections detected with MALS (solid lines, detector voltage). The molar mass (squares) is calculated from the Rayleigh ratio (derived by Astra from the detector voltage) corrected by the UV absorption at 280 nm. The different samples are all concentrated at 100 µM of MIF (WT or C80W) +/- 100 µM of PAV-174 compound. The sample C80W MIF was injected twice to facilitate the calculation molar mass. The trimeric conformation of MIF was not changed upon addition of PAV-174.

Data represents the mean +/- SEM. $^{**}p < 0.01$
